# The pathogenesis of common* Gjb2* mutations associated with human hereditary deafness in mice

**DOI:** 10.1007/s00018-023-04794-9

**Published:** 2023-05-13

**Authors:** Qing Li, Chong Cui, Rongyu Liao, Xidi Yin, Daqi Wang, Yanbo Cheng, Bowei Huang, Liqin Wang, Meng Yan, Jinan Zhou, Jingjing Zhao, Wei Tang, Yingyi Wang, Xiaohan Wang, Jun Lv, Jinsong Li, Huawei Li, Yilai Shu

**Affiliations:** 1 State Key Laboratory of Cell Biology, Shanghai Key Laboratory of Molecular Andrology, Shanghai Institute of Biochemistry and Cell Biology, Center for Excellence in Molecular Cell Science, Chinese Academy of Sciences, Shanghai, China; 2 ENT Institute and Department of Otorhinolaryngology, Eye and ENT Hospital, State Key Laboratory of Medical Neurobiology and MOE Frontiers Center for Brain Science, Fudan University, Shanghai, China; 3 Institutes of Biomedical Sciences, Fudan University, Shanghai, China; 4 NHC Key Laboratory of Hearing Medicine, Fudan University, Shanghai, China; 5 School of Life Science and Technology, Shanghai Tech University, Shanghai, China; 6 School of Life Science, Hangzhou Institute for Advanced Study, University of Chinese Academy of Sciences, Hangzhou, China; 7 Renerval Biotherapeutics, Jiangsu, China

**Keywords:** CX26, CX30, 35delG, 235delC, Hearing loss, Hair cell development, Gap junction channels

## Abstract

Mutations in *GJB2* (Gap junction protein beta 2) are the most common genetic cause of non-syndromic hereditary deafness in humans, especially the 35delG and 235delC mutations. Owing to the homozygous lethality of *Gjb2* mutations in mice, there are currently no perfect mouse models carrying *Gjb2* mutations derived from patients for mimicking human hereditary deafness and for unveiling the pathogenesis of the disease. Here, we successfully constructed heterozygous *Gjb2*^+*/35delG*^ and *Gjb2*^+*/235delC*^ mutant mice through advanced androgenic haploid embryonic stem cell (AG-haESC)-mediated semi-cloning technology, and these mice showed normal hearing at postnatal day (P) 28. A homozygous mutant mouse model, *Gjb2*^*35delG/35delG*^, was then generated using enhanced tetraploid embryo complementation, demonstrating that GJB2 plays an indispensable role in mouse placenta development. These mice exhibited profound hearing loss similar to human patients at P14, i.e., soon after the onset of hearing. Mechanistic analyses showed that *Gjb2* 35delG disrupts the function and formation of intercellular gap junction channels of the cochlea rather than affecting the survival and function of hair cells. Collectively, our study provides ideal mouse models for understanding the pathogenic mechanism of DFNB1A-related hereditary deafness and opens up a new avenue for investigating the treatment of this disease.

**Supplementary Information:**

The online version contains supplementary material available at 10.1007/s00018-023-04794-9.

## Introduction

Congenital hearing loss is one of the most prevalent sensory disorders in children, and more than half of all the cases are caused by genetic mutations [1, 2]. More than 100 genes are involved in syndromic and non-syndromic hearing loss, including autosomal dominant loci, autosomal recessive loci, X-linked loci, and mitochondrial loci (http://hereditaryhearingloss.org) [3, 4]. In 75–80% of cases, the children are born with autosomal recessive non-syndromic hearing loss while both parents have normal hearing [1]. One of the main forms of non-syndromic hearing impairment is autosomal recessive deafness 1A (DFNB1A), which is caused by mutations in *GJB2*, including missense, nonsense, frameshift, insertion, and deletion mutations [5], and such mutations are especially common in the US, many European countries, Israel, Australia, and several Asian countries [5–9]. The c.35delG (also known as c.30delG) and c.235delC alleles are the most common hearing loss-associated *GJB2* alleles in the Caucasian and Asian populations, respectively [5, 7, 8, 10, 11]. The study of the genetic origins and pathophysiological phenotypes of congenital hearing loss will help to decipher the pathogenesis of the disease and promote the development of effective treatments.

The *GJB2* gene encodes gap junction protein 26 (connexin 26, CX26), which is expressed in the non-sensory cells (epithelial cells and supporting cells) of the cochlea assembles with *GJB6* gene encoding connexin 30 (CX30) to form intercellular gap junction channels involved in recycling of potassium ions, and exchange of compounds in the cochlea [12–14]. Gap junction proteins in the cochlear supporting cells are hypothesized to allow rapid removal of K^+^ away from the base of hair cells, resulting in recycling back to the endolymph, and these channels ensure the propagation of Ca^2+^ waves in response to inositol 1,4,5-trisphosphate and possibly to other secondary messengers for mechanoelectrical transduction [15, 16]. To further elucidate the mechanisms of GJB2 in normal auditory function, several mouse models of *Gjb2* mutants have been reported to mimic the pathological phenotypes of human hereditary deafness, including conditional null alleles and transgenic point mutations [17–19]*.* The homozygous mutation in *Gjb2* caused embryo lethality, possibly due to placental defects, which may need to be demonstrated by tetraploid embryo compensation [20–22]. Until now, only the R75W mutation from human patients has been mimicked in a transgenic mouse model that displayed profound hearing loss [19]. However, the R75W mice were established by integrating cDNA sequences carrying R75W into the genome of wild-type mice, so the heterozygous mouse model does not provide accurate material for the study of the pathogenesis and treatment of deafness, and other models, especially those carrying high-frequency pathogenic mutations in populations, such as 35delG or 235delC, need to be established. Thus, the mouse models developed here of homozygous 35delG mutations in *Gjb2* might completely mimic human hereditary deafness and might lead to the identification of pathogenic mechanisms and might provide a good system for testing treatments such as gene therapy.

To overcome the low efficiency of conventional zygote injection for the construction of knock-in mouse models in embryonic-lethal genes (Supplemental Fig. S1), we used enhanced androgenetic haploid embryonic stem cells (AG-haESCs) semi-cloning technology, which carries both *H19*-DMR and *IG*-DMR deletions (termed DKO-AG-haESCs) [23, 24], combined with the CRISPR-Cas9 system enable the rapid construction of heterozygous mouse models related to lethal genes [25, 26]. Thus, in this study we first generated 35delG and 235delC heterozygous mice by semi-cloning technology and then successfully obtained 35delG homozygous mice through tetraploid embryo complementation, which has shown that mouse ESCs alone are capable of supporting embryonic development and growth of mouse, creating a non-chimeric mouse [27]. These models demonstrate that GJB2 plays an important role in mouse and human hearing development and suggest the reasons for hearing loss in patients with *GJB2* mutations.

## Results

### *Gjb2* 35delG and 235delC heterozygous mice show normal hearing at a young

**Fig. 1:**
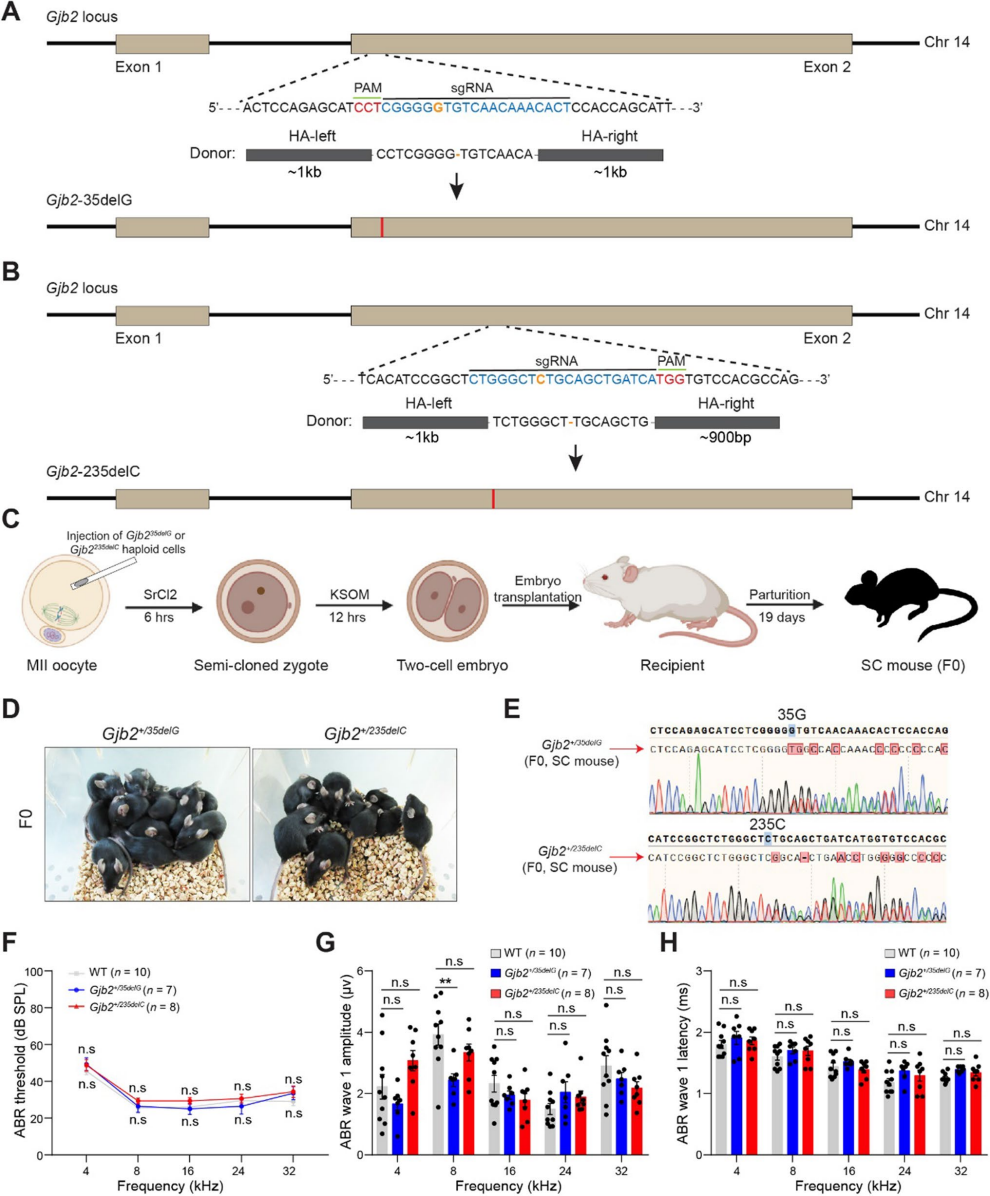
The generation of *Gjb2* 35delG and 235delC heterozygous mice. **A** and **B** Schematic diagram of the construction of 35delG (**A**) and 235delC (**B**) knock-in in *Gjb2*. The blue bases represent the sgRNA sequence. The PAM sequence and the deleted base are shown in red and orange, respectively. The donor sequence ligates to the pMD19T vector. **C** Experimental procedures for producing heterozygous mouse models carrying 35delG or 235delC of *Gjb2* through semi-cloning technology. **D** Images of *Gjb2*^+*/35delG*^ and *Gjb2*^+*/235delC*^ semi-cloned (SC) mice at six weeks. **E** Representative Sanger sequencing results of *Gjb2*^+*/35delG*^ and *Gjb2*^+*/235delC*^ SC mice. **F** The averaged ABR thresholds of the 4-week-old wild-type, *Gjb2*^+*/35delG*^, and *Gjb2*^+*/235delC*^ mice at 4, 8, 16, 24, and 32 kHz. *n* represents the number of tested mice. These heterozygous mice are the offspring of multiple generations of backcrossed with C57BL/6J mice. Wild-type and heterozygous mice from the same litter. **G** and **H** The ABR wave I amplitudes (**G**) and latencies (**H**) of the 4-week-old wild-type, *Gjb2*^+*/35delG*^, and *Gjb2*^+*/235delC*^ mice at 4, 8, 16, 24, and 32 kHz. Data are shown as the mean ± s.e.m of the indicated biological replicates. n.s, no significance, **P < 0.01 by Student’s unpaired two-sided *t*-test

DKO-AG-haESCs combined with CRISPR-Cas9 can efficiently generate knock-in mouse models [28]. We first constructed homologous recombinant vectors containing 35delG and 235delC, respectively (Fig. 1A, B). Then we transfected O48 cells, a DKO-AG-haESC line [24], with pX330-mCherry-35delG and pX330-mCherry-235delC plasmids expressing *Cas9* and sgRNAs targeting exon 2 of *Gjb2* and corresponding donor vectors with 35delG and 235delC mutations, respectively (Fig. 1A, B). After 24 h, mCherry-positive cells with a haploid karyotype were enriched by fluorescence-activated cell sorting (FACS) for single-cell expansion (Supplemental Fig. S2A, B). After 7 days of culture, single clones from the single-cell expansion were picked and lysed for genotyping (Supplemental Fig. S2A). Finally, DKO-AG-haESCs carrying 35delG and 235delC mutations were established (Supplemental Fig. S2C, D).

Next, we injected *Gjb2*^*35delG*^ and *Gjb2*^*235delC*^ haploid cells into wild-type MII oocytes and successfully obtained heterozygous semi-cloned mice carrying the 35delG or 235delC mutation in *Gjb2* (Fig. 1C–E). Female F0 semi-cloned mice were backcrossed with wild-type C57BL/6J males for more than five generations to exclude the potential influence of *H19*-DMR and *IG*-DMR deletions and off-targets (Supplemental Fig. S3A, B). Bodyweight analysis showed that heterozygous mutation of 35delG or 235delC did not affect the growth of mice (Supplemental Fig. S3C, D). Auditory brainstem response (ABR) tests also showed that the hearing thresholds of *Gjb2*^+*/35delG*^ and *Gjb2*^+*/235delC*^ knock-in mice were similar to wild-type mice at frequencies of 4, 8,16, 24, and 32 kHz at postnatal day 28 (P28) (Fig. 1F–H). These results demonstrated that younger mice carrying heterozygous 35delG and 235delC mutations showed normal ontogeny and auditory function, similar to that of human carriers [29].

### *Gjb2* 35delG homozygous mice show complete hearing loss at P35

**Fig. 2:**
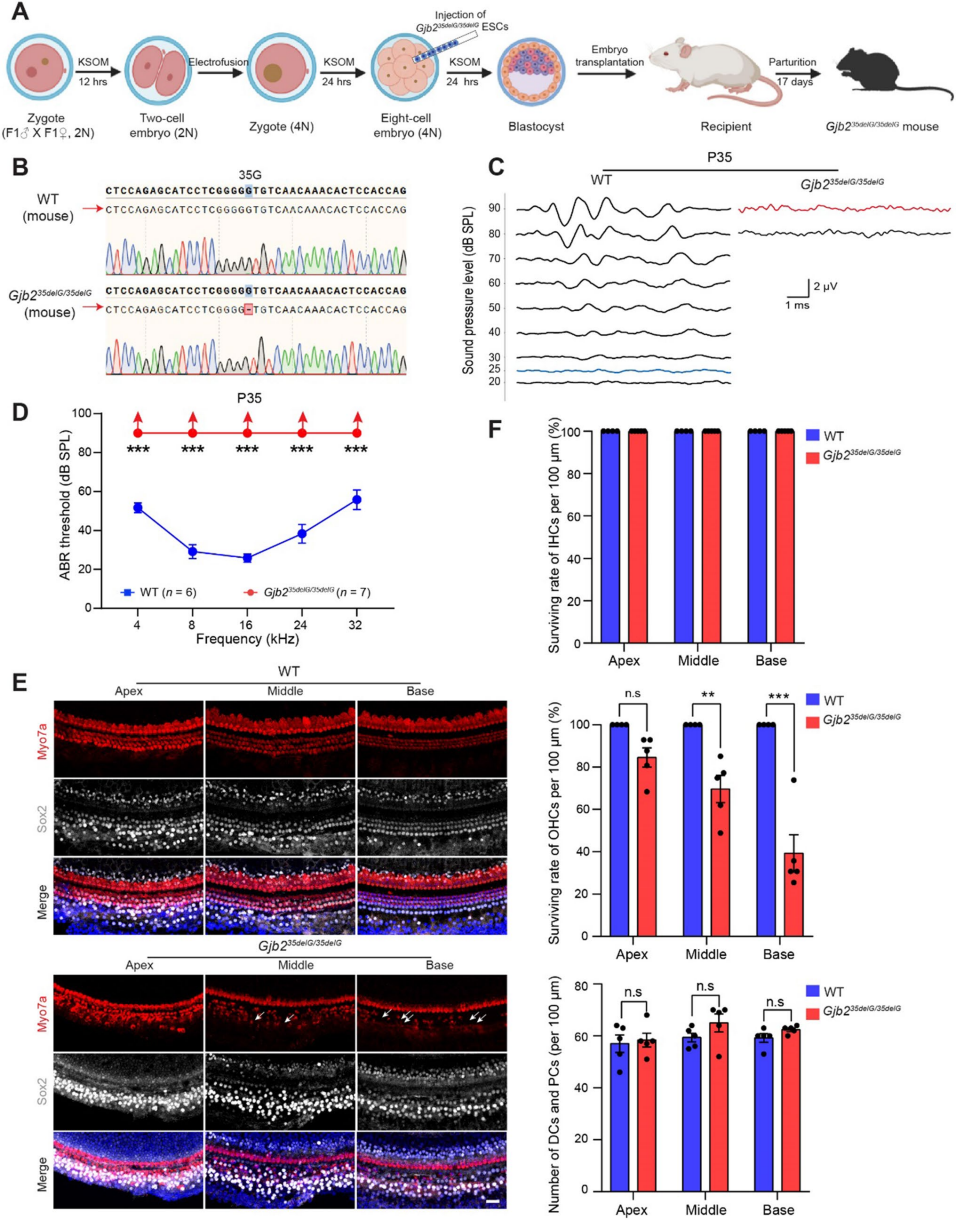
The generation of *Gjb2* 35delG homozygous mice with profound deafness. **A** Schematic diagram of the generation of *Gjb2* 35delG homozygous mice via tetraploid embryo complementation (created in BioRender.com). **B** Representative Sanger sequencing results of wild-type and *Gjb2*^*35delG/35delG*^ males. **C** ABR waveforms were recorded at 8 kHz in the wild-type and *Gjb2*^*35delG/35delG*^ mice at P35. The blue and red traces indicate the thresholds of wild-type and *Gjb2*^*35delG/35delG*^ males, respectively. The scale bar for all traces is shown. **D** The average ABR thresholds of the P35 wild-type and *Gjb2*^*35delG/35delG*^ mice at 4, 8, 16, 24, and 32 kHz. Upward arrows indicate that there were no ABR responses at 90 dB SPL for the corresponding frequencies, and the hearing threshold was recorded as 90 dB SPL. *n* represents the number of tested mice. **E** Representative confocal microscopy images of Myo7a and Sox2 immunofluorescence in the apical, middle, and basal turns of the cochleae from wild-type and *Gjb2*^*35delG/35delG*^ mice at P35. White arrows indicate the loss of hair cells. Scale bar, 20 μm. **F** Quantification of surviving IHCs, OHCs, supporting cells of Deiters’ cells and outer/inner pillar cells (DCs and PCs) in the wild-type and *Gjb2*^*35delG/35delG*^ males at P35, respectively. Individual values are shown. Data are shown as the mean ± s.e.m of the indicated biological replicates. n.s, no significance, **P < 0.01, ***P < 0.001 by Student’s unpaired two-sided t-test

To determine the phenotypes and functional effects caused by complete loss of the GJB2 protein, we tried to obtain mouse models carrying homozygous 35delG or 235delC by mating heterozygotes. No homozygotes were observed and the proportion of heterozygotes did not follow Mendel’s laws (Supplemental Fig. S3E), and this might also be caused by embryonic lethality due to placental defects similar to what was seen for *Gjb2* knock-out mice in previous work [22]. Tetraploid embryo complementation is the gold standard for demonstrating and rescuing placental defects and can result in live births [30–32]. Thus, we first derived embryonic stem cell (ESC) lines carrying homozygous 35delG in *Gjb2* from the inner cell mass of preimplantation blastocysts, which were obtained from in vitro fertilization (IVF) (Supplemental Fig. S4A–C). Previous studies showed that the efficiency of producing ESC-derived mice via injection into 4–8-cell tetraploid embryos is greater than that obtained via tetraploid blastocyst complementation [20, 21]. Thus, we attempted to obtain homozygous mutant mice by injecting about ten *Gjb2*^*35delG/35delG*^ ESCs into one 4–8-cell tetraploid embryo using a piezo manipulator (Fig. 2A). In total, we successfully obtained 26 live pups carrying the homozygous 35delG mutation out of 1719 reconstructed blastocysts in 18 independent experiments (Fig. 2B, Supplemental Fig. S5A and Table S1). Taken together, these results demonstrated that GJB2 plays an indispensable role in mouse placenta development.

To determine whether *Gjb2*^*35delG/35delG*^ mice can mimic human hereditary deafness, we performed ABR tests at frequencies of 4, 8, 16, 24, and 32 kHz on homozygous and wild-type mice at 5 weeks of age. The results showed that *Gjb2*^*35delG/35delG*^ mice had significantly elevated ABR thresholds across all frequencies tested and had completely lost their hearing (Fig. 2C D), which is consistent with patients carrying homozygous 35delG mutations in the *GJB2* gene [33]. Meanwhile, we found that the homozygous 35delG mutation also had no effect on the growth of the mice (Supplemental Fig. S5A, B). The organ of Corti is the epithelium of the cochlea and contains one row of inner hair cells (IHCs) and three rows of outer hair cells (OHCs), which can convert the pressure fluctuations of sound into electrical signals through mechanoreceptors [34]. Supporting cells, such as Deiters’ cells and pillar cells surrounding sensory hair cells, play an essential role in the physiology of the cochlea and help convey auditory inputs to the brain [35, 36]. Immunofluorescence staining showed that OHCs, but not IHCs, were partially lost in 35delG homozygotes from the apical turn to the basal turn of the cochlea, which damaged frequency selectivity and signal amplification (Fig. 2E, F). However, supporting cells did not show a significant change in the cochlea of the *Gjb2*^*35delG/35delG*^ mice at P35 (Fig. 2E, F). Taken together, hearing loss in the *Gjb2*^*35delG/35delG*^ mice appears to be caused by the degeneration of sensory hair cells, which is similar to what is seen in the R75W transgene and *Gjb2* P0 knockdown mouse models [19, 37].

### GJB2 absence disrupts the GJCs formation of the cochlea

In previous studies, *Gjb2*^*loxP/loxP*^*;Rosa26*^*CreER*^ mice were injected with 4-hydroxytamoxifen to knockdown *Gjb2* at different time points after birth, including P0, P5, P6, P8, P10, P12, P15, and P20, and this showed that *Gjb2* knock-down before P8 caused profound hearing loss, failure of the tunnel of Corti to open up, and massive hair cell degeneration [37–39], indicating that GJB2 plays important roles in the postnatal maturation of the cochlea. Immunostaining showed that GJB2 was localized in the cochlea from embryonic day 16 (E16) [40]. To reveal the function of GJB2 in the cochlea during embryonic development, we first analyzed the supporting cells and hair cells at E17.5 and P0 using immunofluorescence. The results showed that the number and distribution of supporting cells and hair cells were unaffected in the apical, middle, and basal turns of the cochlea at E17.5 and P0 when *Gjb2* was disrupted (Supplemental Fig. S6A, B). Moreover, scanning electron microscopy (SEM) showed that the structures of hair cell stereocilia in *Gjb2*^*35delG/35delG*^ mice were also normal compared to wild-type mice during embryonic development (Supplemental Fig. S6C, D). These results indicated that *Gjb2* is dispensable for the prenatal development of hair cells and supporting cells in the cochlea.

**Fig. 3:**
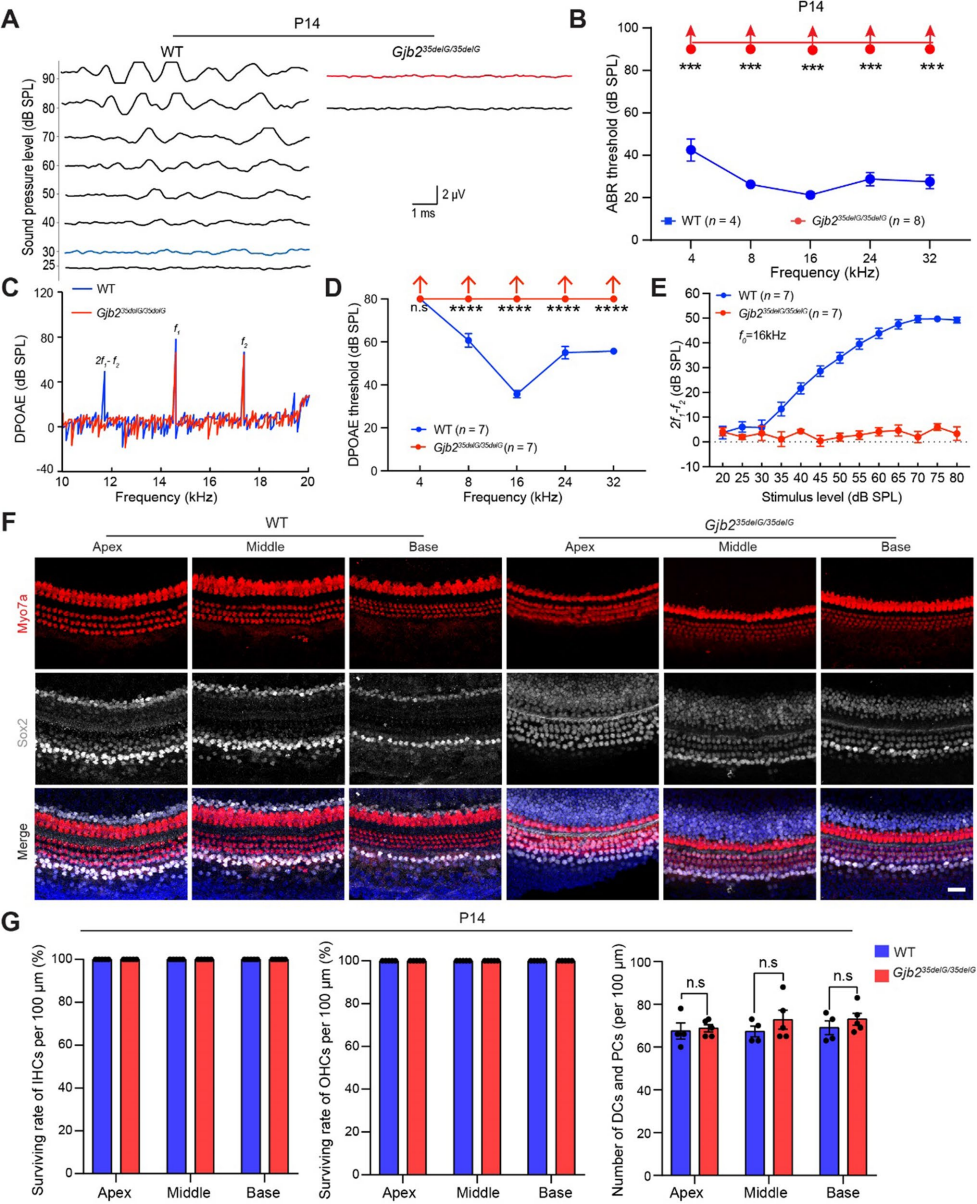
The influence of GJB2 in the cochlea at P14. **A** ABR waveforms were recorded at 8 kHz in the wild-type and *Gjb2*^*35delG/35delG*^ males at P14. The blue and red traces indicate the thresholds of wild-type and *Gjb2*^*35delG/35delG*^ mice, respectively. The scale bar for all traces is shown. **B** The average ABR thresholds of the P14 wild-type and *Gjb2*^*35delG/35delG*^ males at 4, 8, 16, 24, and 32 kHz. Upward arrows indicate that there were no ABR responses at 90 dB SPL for the corresponding frequencies, and the hearing threshold was recorded as 90 dB SPL. *n* represents the number of tested mice. Data are shown as the mean ± s.e.m. *** *P* < 0.001 by Student’s unpaired two-sided *t*-test. **C** Spectra of DPOAE were recorded from the wild-type (blue trace) and *Gjb2*^*35delG/35delG*^ (red trace) males at P14. *f*_0_ = 16 kHz, *f*2/*f*1 = 1.2, *L*_1_/*L*_2_ = 60/60 dB SPL. The peak of DPOAE (2*f*_1_–*f*_2_) in *Gjb2*^*35delG/35delG*^ mice was not available but *f*1 and *f*2 peaks remained the same as those in wild-type mice. **D** The average DPOAE thresholds were measured on the same animals performed with ABR recording at 4, 8, 16, 24, and 32 kHz. Upward arrows indicate that there were no DPOAE responses at the stimulus level of 80 dB SPL for the corresponding frequencies, and the DPOAE threshold was recorded as 80 dB SPL. *n* represents the number of tested mice. Data are shown as the mean ± s.e.m. **** *P* < 0.0001 by Student’s unpaired two-sided *t*-test. **E** Input/output function of the DPOAE (2*f*_1_–*f*_2_) amplitudes of wild-type and *Gjb2*^*35delG/35delG*^ males at 16 kHz frequency at P14. Data are shown as the mean ± s.e.m. The dotted line is the noise level. **F** Representative confocal microscopy images of Myo7a and Sox2 immunofluorescence in the apical, middle, and basal turns of the cochleae from wild-type and *Gjb2*^*35delG/35delG*^ males at P14. Scale bar, 20 μm. **G** Quantification of surviving inner hair cells (IHCs), outer hair cells (OHCs), supporting cells of Deiters’ cells and outer/inner pillar cells (DCs and PCs) in the wild-type an*d Gjb2*^*35delG/35delG*^ males at P14, respectively. Data are shown as the mean ± s.e.m of the indicated biological replicates. n.s., no significance by Student’s unpaired two-sided *t*-test

The mouse cochlea is not fully developed at birth and it continues to mature until P14, at which point hearing becomes active, so we tested the ABR of *Gjb2*^*35delG/35delG*^ mice at P14. As expected, the homozygous mice were completely deaf at P14 at frequencies of 4, 8, 16, 24, and 32 kHz (Fig. 3A, B). Subsequently, we performed distortion product otoacoustic emission (DPOAE) reflecting the active cochlear amplification to assess fine changes in OHCs function. We found that the DPOAEs could not be induced in the 35delG homozygous mice with profound deafness (Fig. 3C–E), indicating that the function of OHCs is damaged. Besides, *Gjb2*^*35delG/35delG*^ mice exhibited the delayed apoptosis of greater epithelial ridge (GER) cells and GER retention (Supplemental Fig. S7A), which is similar to the previous study [41]. To our surprise, the hair cells and supporting cells around the hair cells were also intact in the homozygous mice (Fig. F, G), indicating that the degeneration of hair cells at P35 in the cochlea was not the direct cause for hearing loss in 35delG homozygous mice.

**Fig. 4:**
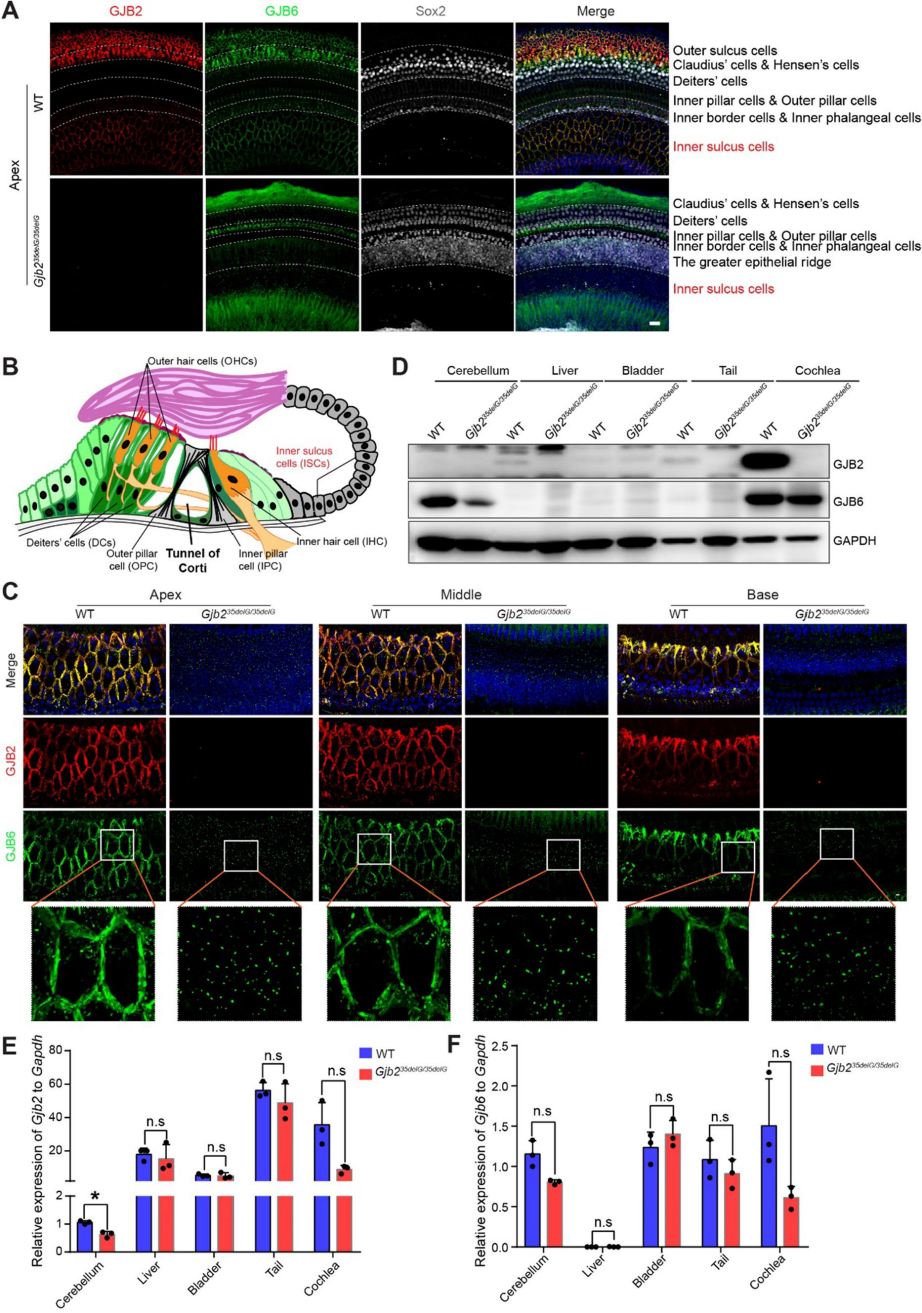
The mutation of GJB2 disrupts the formation of GJCs by reducing the GJB6 protein. **A** The panoramic view of representative confocal images of apical turns from wild-type and 35delG homozygous mice at P14, by staining GJB2, GJB6, and Sox2. The regions containing different supporting cells were delineated in the figure. Scale bar, 20 μm. **B** The diagram of the organ of Corti. **C** Representative confocal microscopy images of GJB2 and GJB6 immunofluorescence in the apical, middle, and basal turns of the cochleae from wild-type and *Gjb2*^*35delG/35delG*^ mice at P14. The inner sulcus areas were shown. Three independent mice were analyzed for each group. Scale bar, 20 μm. The bottom row shows high magnification views of GJB6. **D** Western blots showing GJB2 and GJB6 protein expression levels in the different tissues of wild-type and *Gjb2*^*35delG/35delG*^ mice, including cerebellum, liver, bladder, tail, and cochlea. Results obtained with GADPH are shown as controls for the amount of protein loading in each lane. Two biological repeats for each group. **E** and **F** Transcriptional analysis of *Gjb2* (**E**) and *Gjb6* (**F**) genes in different tissues of wild-type and *Gjb2*^*35delG/35delG*^ mice by qPCR, including cerebellum, liver, bladder, tail, and cochlea. Three biological repeats for each group. The expression values were normalized to that of *Gapdh*. Data are shown as the average mean ± s.e.m. *P < 0.05; n.s, no significant difference

GJB2 and GJB6 form the intercellular gap junction channels (GJCs) of non-sensory cells in the cochlea [13, 42]. *Gjb6* knock-out mice show profound hearing loss with a reduction of GJB2 protein, which disrupts the formation of GJCs [12, 43]. Thus, we first determined the integrity of the GJCs by immunofluorescence of GJB2 and GJB6 in the whole cochlea at P14. As expected, GJB2 was not observed in 35delG homozygous mice (Fig. 4A). However, to our surprise, GJB6 protein was significantly down-regulated in the supporting cells of 35delG homozygous mice resulting in the total disruption of GJCs, suggesting that the macromolecular complex had been degraded (Fig. 4A and Supplemental Fig. S8A). In a detailed analysis of the GJCs structure in the inner sulcus cells (ISCs), we observed that in wild-type cochleae, the GJCs were extensive and flat at the cell periphery, arranged in neat pentagonal or hexagonal shapes, while in 35delG homozygous cochleae, the GJCs were considerably broken down and appeared as tiny, vesicle-like structures (Fig. 4B, C). We observed that GJC disruption was already present in P0 mice (Supplemental Fig. S8B), indicating the impairment of the cochlea prior to the onset of hearing. We further analyzed the protein expression of GJB2 and GJB6 in different tissues (cerebellum, liver, bladder, tail, and cochlea) between wild-type and *Gjb2*^*35delG/35delG*^ mice at P14 by western blot. The result showed that GJB6 was comparable to wild-type bladder and tail in 35delG homozygous mice with a low expression, but was reduced in cerebellum and cochlea with a high expression (Fig. 4D). To answer the question of whether the loss of GJB6 protein is due to transcriptional regulation, the qPCR results showed that the expression of *Gjb2* and *Gjb6* in 35delG homozygotes was also reduced in cerebellum and cochlea (Fig. 4E, F). Taken together, these results suggested that GJB2 and GJB6 expression is interrelated at the transcription and translation levels in the cerebellum and cochlea, which may be regulated through the NF-κB pathway and contribute to deafness [12].

### The 35delG mutation in *Gjb2* impair structure and function of the cochlea

**Fig. 5:**
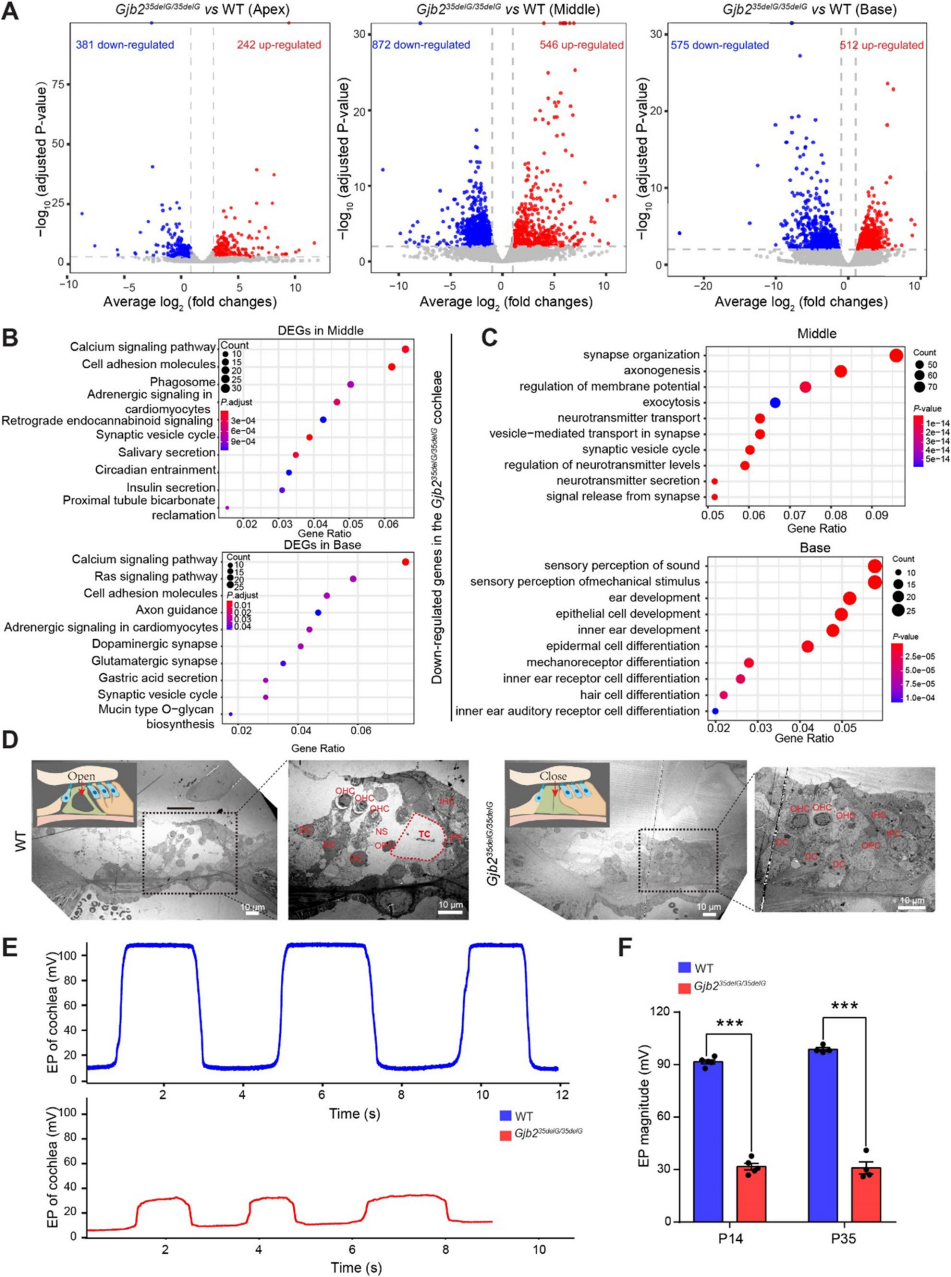
The disruption of GJCs in homozygous 35delG impairs the structure and function of cochlea. **A** Volcano plot showing the DEGs between *Gjb2*^*35delG/35delG*^ and wild-type cochleae in the apical, middle, and basal turns (adjusted *P*-value < 0.01 and fold changes > 2). **B** The KEGG pathway analysis of DEGs between wild-type and *Gjb2*^*35delG/35delG*^ cochleae in the middle and basal turns. **C** GO analysis of down-regulated genes between *Gjb2*^*35delG/35delG*^ and wild-type cochleae in the middle and basal turns. **D** Representative TEM images of the tunnel of Corti in the middle turn of the cochleae from wild-type and *Gjb2*^*35delG/35delG*^ mice at P14. The right images show the high magnification (1400 × magnification) views of the left black box (460 × magnification). Two independent mice were analyzed for each group. Scale bar: 10 μm. TC, tunnel of Corti; NS, Nuel's space; IHC, inner hair cell; OHC, outer hair cell; IPC, inner pillar cell; OPC, outer pillar cell; DC, Deiters’ cell. **E** Representative EP recordings from the cochleae of wild-type and *Gjb2*^*35delG/35delG*^ mice at P35. **F** The average EP magnitudes from the cochleae of wild-type and *Gjb2*^*35delG/35delG*^ mice at P14 and P35. Data are shown as the mean ± s.e.m of the indicated biological replicates. n.s, no significance, ***P < 0.001 by Student’s unpaired two-sided *t*-test

To investigate the effects of GJCs abnormalities on inner ear cells, we performed RNA-seq in the cochlea at P14 to investigate the cause of hearing loss in homozygous 35delG mice. The cochlea was surgically divided into the apical, middle, and basal turns according to the sensitivity to different sound frequencies for RNA extraction, library construction, and sequencing (Supplemental Fig. S9A). Principal component analysis of the transcriptome data showed a similarity between repeat samples, but a clear difference between wild-type and homozygous mice, especially in the middle and basal turns, which was consistent with the immunofluorescence results (Fig. 2E and Supplemental Fig. S9B). Differential gene expression analysis (adjusted *P*-value < 0.01 and fold changes > 2) also showed that more differentially expressed genes (DEGs) appeared in basal and middle turns (623 DEGs in the apex, 1418 DEGs in the middle, and 1087 DEGs in the base) (Fig. 5A). The Kyoto Encyclopedia of Genes and Genomes (KEGG) pathway analysis revealed that the DEGs of the apical turn were enriched in the PI3K-AKT signaling pathway and synaptic vesicle cycle (Supplemental Fig. S9C), both of which are essential for hearing acquisition [12, 44]. DEGs in the middle and basal turns were closely associated with Ca^2+^ signaling pathways, cell adhesion molecules, and synaptic vesicle cycle (Fig. 5B). Cell adhesion molecules contribute to regionalization in the otocyst, cellular differentiation, and neurite extension to ensure proper inner ear and hair cell development, and Ca^2+^ signaling pathways control the mechano-transduction and synaptic transmission in sensory hair cells, and disrupting either of these pathways can lead to non-syndromic deafness [45, 46]. Gene ontology (GO) analysis further showed that down-regulated genes were also enriched in synapse organization, axonogenesis, regulation of membrane potential, and synaptic vesicle cycle in both the apical and middle turns (Fig. 5C and Supplemental Fig. S9D), all of which are essential processes for hearing transduction, transmission, and acquisition [45, 46]. Genes related to the sensory perception of sound and inner ear development were significantly down-regulated in the basal turn, including *Myo3a*, *Myo3b*, *Myo15*, *Otof*, *Tmc1*, etc., (Fig. 5C). Taken together, these results suggest that *Gjb2*^*35delG/35delG*^ may impair cochlear function by reducing the propagation of Ca^2+^ signaling and disrupting synapse organization.

GJCs recirculate K^+^ ions from hair cells to the marginal cells of the stria vascularisir and allow the diffusion of the Ca^2+^-mobilizing secondary messenger inositol 1,4,5-trisphosphate across coupled cells [13, 42]. Besides, the gap junctions linking epithelial cells are important to create the tunnel of Corti, which contributes to the secretion of K^+^ ions into endolymph [19, 47]. Then, the endocochlear potential (EP), which is primarily responsible for driving cations (mostly K^+^) from the endolymph into the sensory hair cells through mechanoelectrical transduction channels and thus is essential to hearing, is severely impaired in *Gjb6*-deficient mice [43, 48]. We revealed that the loss of GJCs disrupted the propagation of intercellular Ca^2+^ signals and synaptic transmission by RNA-seq analysis (Fig. 5B and Supplemental Fig. S9C). In addition, by conducting a three-dimensional analysis of confocal images, it was revealed that 35delG homozygous mice exhibited a failure of the tunnel of Corti to open (Supplemental Fig. S9E). In order to analyze the state of the tunnel of Corti in detail, we employed both light microscopy and transmission electron microscopy (TEM) imaging techniques and found that the tunnel of Corti completely collapses in 35delG homozygous mice (Fig. 5D and Supplemental Fig. S9F), which is similar to R75W transgene and P0 knockdown models [19, 39]. Furtherly, we measured the EP of wild-type and 35delG homozygous mice at P14 and P35 and found a significant reduction of EP in 35delG homozygous mice at both ages, thereby directly contributing to the profound deafness (Fig. 5E, F). Collectively, these results suggest that the loss of GJB2 impairs the structure and function of the cochlea, and restoring the function of these channels might rescue hearing loss in mice [49].

## Discussion

Mutations in *GJB2* are the main genetic factor leading to congenital hearing loss, especially the 35delG and 235delC mutations [5]. To determine the pathogenic mechanisms and promote disease treatment, it is important to construct disease-related mutation mouse models. To date, only the R75W transgene and *Gjb2* conditional knock-out mouse models have been generated due to the embryonic lethality of homozygous *Gjb2* inactivation and hindered the pathogenic mechanism exploration to some degree [17, 19, 22]. In this study, we successfully constructed *Gjb2* 35delG and 235delC heterozygous mice through AG-haESC-mediated semi-cloning technology, showing that heterozygotes had normal ontogeny and auditory function. Our versatile approach has the unique advantage over conventional zygote injection or electroporation for the construction of knock-in mouse models of embryonic-lethal genes (Supplemental Fig. S1). AG-haESCs enable the rapid generation of knock-in cell lines of embryonic-lethal genes in vitro, and heterozygous knock-in mice can be obtained by intracytoplasmic injection (ICAHCI) in a single generation (Supplemental Fig. S1). In contrast, CRISPR-Cas9-mediated homology-directed repair for producing knock-in mice results in high proportions of indels or mosaic mice [50, 51], which may be lethal in the embryonic stage or soon after birth. Thus, it is very hard to generate knock-in mouse models of embryonic-lethal genes via zygote injection or electroporation.

Next, in an attempt to overcome the failure of placental development in *Gjb2* homozygous mutant embryos, we first generated *Gjb2*^*35delG/35delG*^ ESCs and then obtained *Gjb2*^*35delG/35delG*^ mice through tetraploid embryo complementation. In this study, we provide direct evidence that defects in the placenta underlie the failure of *Gjb2* homozygous mutant embryos. However, we were able to obtain live *Gjb2*^*35delG/35delG*^ mice with a low success rate (116/1403, 8.3% of reconstituted embryos), and a few of these offspring survived to adulthood (25/116, 21.6% of live offspring) (Supplemental Table S1). The survival rate of ESC-derived mice is extremely low owing to the poor genetic heterozygosity and fitness of tetraploid embryos, the genetic background of the ESCs, and the operational approach of tetraploid complementation [20, 31, 32, 52]. Thus, improving the survival rate of *Gjb2*^*35delG/35delG*^ mice will require optimizing the genetic background of the tetraploid embryo and testing different experimental conditions.

The 35delG mutation is the most common disease-causing *Gjb2* mutation in Europe, North America, and South America, whereas the 235delC mutation is the most common in Asia [5]. Our study first established 35delG and 235delC heterozygous mouse models that are useful for mimicking and studying presbycusis, which occurs in heterozygous human carriers of 35delG and 235delC, rather than just being a substitute for *Gjb2*^+/−^ mice (exon 2 deletion) [29, 53]. We then successfully generated a 35delG homozygous mouse model that showed profound hearing loss and completely mimicked human hereditary deafness caused by 35delG homozygous mutation. Although *Gjb2* is expressed in the cochlea from E16 [40], our immunostaining and scanning electron microscopy results suggested that the supporting cells and hair cells of homozygous 35delG mice developed normally at E17.5, P0, and P14, indicating that GJB2 is not essential for the survival of cochlear cells during early developmental stages. However, the outer hair cells, not the supporting cells, were significantly degenerated at P35, especially at the basal turn in 35delG homozygous mice. Besides, we also found that the GJCs were completely disrupted in 35delG homozygous mice with reduced GJB6 protein from P0, resulting in the failure of K^+^ recycling and Ca^2+^ signaling. The RNA-seq analysis and EP measurement strongly supported these assumptions, indicating the key mechanisms for GJB2-mediated hearing acquisition.

To study the pathogenesis of hereditary deafness caused by *Gjb2* mutations, only an R75W transgenic and some conditional knock-out mouse models (including Cx26^*OtogCre*^, Cx26^*Sox10Cre*^, P0 knockdown, etc.) have been established, but there are not typical disease models of non-syndromic deafness caused by *Gjb2* mutations [18, 19, 42, 54, 55]. In comparison with these mouse models, our 35delG homozygous mice showed complete deafness with intact hair cells and supporting cells at P14, but R75W, Cx26^*Sox10Cre*^, and P0 knockdown mouse models showed more severe hearing loss than Cx26^*OtogCre*^ model, most likely a result of the different space-specific and time-specific methods used to generate these mice [18, 19, 42, 54, 55]. Important similarities in cochlear pathology exist between the current and previously reported mutant mice. Cx26^*OtogCre*^ mice also showed that GJB6 expression was developmentally delayed at P6 and P30 [55], which may destroy GJCs formation similar to our model. R75W, Cx26^*OtogCre*^, and Cx26^*Sox10Cre*^ models, but not P0 knockdown mice, also showed failure in the formation of the tunnel of Corti [18, 19, 54, 55], indicating that GJB2 is essential to maintain the structure of cochlear duct. Our results showed that 35delG homozygous mice significantly reduced EP at P14, and Cx26^*OtogCre*^ and Cx26^*Sox10Cre*^ mice had a significantly decreased EP compared to control mice at adult, whereas R75W mice harbored normal EP at adult [18, 19, 55]. Besides, Cx26^*Sox10Cre*^-derived cultured cochlea showed fail to propagate the Ca^2+^ signals, which was similar to our RNA-seq results of 35delG homozygous cochlea, indicating that GJB2 is necessary for intercellular calcium signaling activity [42]. Some differences between these models warrant more investigation. Our 35delG homozygous mice showed profound deafness with intact hair cells and supporting cells at P14, i.e., soon after the onset of hearing, but R75W, Cx26^*OtogCre*^, and P0 knockdown mice showed initial degeneration of hair cells and supporting cells at P14 [18, 19, 54]. Thus, our results indicated that the degeneration of hair cells and supporting cells in the *Gjb2* mutant cochlea is not the major cause of hearing loss, but the disruption of GJCs caused low EP and defect of Ca^2+^ responses may directly lead to failure of the hearing acquisition.

In summary, our study demonstrated the function of GJB2 in cochlear development from embryos to adults, thus suggesting an avenue for the treatment of DFNB1A patients such as restoration of GJCs through overexpression of GJB2 or GJB6 [49, 56] or correction of mutant *GJB2* through postnatal genome editing rather than through germ cell editing [57–60]. The mouse model generated in this study would provide a valuable tool for investigating therapeutic means to restore the GJCs in the organ of Corti at an early stage of development.

## Materials and methods

### Mice

All animal procedures were approved by the Institutional Animal Care and Use Committee of the Center for Excellence in Molecular Cell Science and the Institutional Animal Care and Use Committee of Fudan University. Mice were housed in individually ventilated cages in an accredited specific pathogen-free facility and a 12 h dark–light cycle. MII oocytes were collected from female B6D2F1 (C57BL/6J♀ × DBA2♂) mice. All the pseudopregnant foster mothers were ICR females. The genetic background of *Gjb2*^*35delG/35delG*^ males established in this study by tetraploid complementation assay was almost C57BL/6J. Wild-type males of the same age as controls were purchased from Shanghai SLAC laboratory animal Co. Ltd.

### Plasmid construction

For the construction of CRISPR-Cas9 plasmids, sgRNAs targeting *Gjb2* (termed pX330-mCherry-35delG and pX330-mCherry-235delC, respectively) were designed, synthesized, annealed, and ligated to the pX330-mCherry plasmid [61], which was digested with Bpi I (Thermo Scientific, Cat# FD1014). For the construction of donor plasmids (termed 19T-Gjb2-35delG and 19T-Gjb2-235delC), the sequences of the left and right homologous arms with 35delG and 235delC were amplified from the genome using Phanta Max Super-Fidelity DNA Polymerase (Vazyme, Cat# P505). The left/right arms of 35delG and 235delC were obtained by overlap PCR with mixed templates and then ligated to the pMD19T vector. All plasmids were collected using the EndoFree Maxi Plasmid Kit (TIANGEN, Cat#DP117) and were confirmed by Sanger sequencing. The oligonucleotide sequences used for plasmid construction in this study are listed in Supplemental Table S2.

### Cell culture and transfection

Mouse DKO-AG-haESCs (*IG*^*ΔDMR*^-*H19*^*ΔDMR*^-AGH-2 cells or O48 cells) [24] were cultured in ESC medium containing 15% FBS (Excell Bio), penicillin–streptomycin, non-essential amino acids, Nucleosides, l-glutamine, β-mercaptoethanol, 1000 U/ml Lif, 1 μM PD03259010 (Selleck), and 3 μM CHIR99021 (Selleck) in DMEM (Gibco) at 37 ℃ and 5% CO_2_. As previously described [23, 24], DKO-AG-haESCs were passaged every 2–3 days by trypsinization, and haploid cells were enriched every 2–3 weeks by fluorescence-activated cell sorting (FACS). One million AG-haESCs were passaged into a well of a six-well plate and then transfected with 2 μg CRISPR-Cas9 plasmids (pX330-mCherry-35delG or pX330-mCherry-235delC) and 1 μg pMD19T vectors (19 T-Gjb2-35delG or 19 T-Gjb2-235delC, respectively) using Lipofectamine 3000 (Life Technologies) following the manufacturer’s instructions. After 18–24 h, mCherry-positive haploid cells were enriched by FACS and then 6000–10,000 cells were seeded into a new well of a six-well plate. After 6–8 days, single clones derived from a single cell were picked and genotyped using PCR and Sanger sequencing.

### ICAHCI and embryo transfer

To generate semi-cloned mice, *Gjb2*^*35delG*^ and *Gjb2*^*235delC*^ haploid cells were arrested at M phase by culturing in ESC medium containing 0.05 μg/ml demecolcine for 12 h before ICAHCI (intracytoplasmic AG-haESC injection). Arrested haploid cells were trypsinized and suspended in H-CZB medium for ICAHCI. A single haploid cell was then injected into the cytoplasm of MII oocytes, which were collected from superovulated B6D2F1 females, using a Piezo-drill micromanipulator. The reconstructed zygotes were cultured in CZB medium for 30 min and then activated for 5–6 h in Ca^2+^-free CZB with SrCl_2_. After activation, the reconstructed zygotes were cultured in AA-KSOM (Merk) medium at 37 °C and 5% CO_2_. After 18 h, the semi-cloned embryos reached the two-cell stage and then were transferred into each oviduct (18–23 embryos) of 0.5 day post-coitum (dpc) pseudo-pregnant ICR females.

### Derivation of ESCs

To generate blastocysts to derive *Gjb2*^*35delG/35delG*^ ESCs, zygotes were obtained through IVF. Cumulus-oocyte complexes and sperms were obtained from *Gjb2*^+*/35delG*^ females and males, respectively, and incubated in a drop of HTF (Millipore, Cat#MR-070-D) containing reduced L-glutathione (170 µg/µL) (Sigma, Cat#G4251) for 3 h at 37 °C and 5% CO_2_. The zygotes from IVF cultured in AA-KSOM developed into two-cell embryos after 1 day and formed blastocysts after 4 days. The zona pellucida of the blastocysts was removed using acidic Tyrode’s solution. Each blastocyst was transferred into one well of a 96-well plate seeded with ICR embryonic fibroblast feeders in ESC medium. After 7–10 days of culture, the ESC colonies derived from the inner cell mass of blastocysts were trypsinized and transferred to a 48-well plate without a feeder layer in fresh ESC medium. Clonal expansion of the ESCs proceeded from 48-well plates to 6-well plates without feeder cells and then in 6-well plates for routine culture and genotyping. All derived ESC lines were male.

### Generation of tetraploid embryos

Eight-week-old B6D2F1 females were superovulated with 6 international units of pregnant mare’s serum gonadotropin (PMSG) for 48 h and then injected with human chorionic gonadotropin (hCG), then mated to B6D2F1 males for 12 h. Zygotes were harvested from the oviducts of B6D2F1 females with a vaginal plug after 24 h post-hCG injection using hyaluronidase (Sigma, Cat# H3884). After 12 h, the zygotes developed into two-cell embryos and were electrofused to produce one-cell tetraploid embryos. Two-cell embryos were aligned using an alternating current in 0.3 M mannitol solution, and a single direct current pulse of 1000 V/cm was applied for 20 µs. After electrofusion, the tetraploid embryos were returned to AA-KSOM medium and cultured for one day to reach the 4–8-cell stage.

### Tetraploid embryo injection and blastocyst transfer

To generate *Gjb2*^*35delG/35delG*^ mice, male *Gjb2*^*35delG/35delG*^ ESCs were trypsinized and suspended in KnockOut DMEM (Gibco, Cat# 10,829,018) containing 15% KSR for tetraploid embryo injection. A total of 10–15 ESCs were then injected between the blastomere under the zona pellucida of 4–8-cell tetraploid embryos in a droplet of 15% KSR medium using a Piezo-drill micromanipulator. The reconstructed embryos were cultured in AA-KSOM medium for 24 h at 37 °C and 5% CO_2_ and then transferred into each uterine horn (10–13 embryos) of 2.5 dpc pseudo-pregnant ICR females following standard procedures. On day 19.5 of gestation, the recipients were subjected to cesarean section, and live pups were nursed by lactating ICR females.

### Genotyping and sanger sequencing

The tails and toes of heterozygotes (*Gjb2*^+*/35delG*^ and *Gjb2*^+*/235delC*^ mutant mice) and homozygotes (*Gjb2*^*35delG/35delG*^ mutant mice) were directly lysed by 50 μL lysis buffer from the Mouse Direct PCR Kit (Bimake), incubated at 55 °C for 90 min and 95 °C for 5 min. The lysate (about 2 μL) was used as a PCR template to amplify the mutant site in *Gjb2* using Phanta Flash Master Mix (Vazyme). Products of PCR were purified by gel electrophoresis using Universal DNA Purification Kit (TIANGEN) and then performed Sanger sequencing. The primer sequences are listed in Table S2.

### RNA extraction and quantitative real-time PCR (qPCR)

Total RNA was isolated from the different tissues (cerebellum, liver, bladder, tail, and cochlea), which were cut with scissors, of wild-type and *Gjb2*^*35delG/35delG*^ mice at P14 using TRIzol reagent (Invitrogen). The cDNA was obtained from about 1 µg RNA with reverse transcription reaction by the HiScript III 1st Strand cDNA Synthesis Kit (+ gDNA wiper) (Vazyme). Quantitative real-time PCR reactions were performed on a qTOWER^3^ (Analytik Jena) using the ChamQ Universal SYBR qPCR Master Mix (Vazyme) in triplicate. All gene expression was calculated based on the 2^−∆∆Ct^ method after normalization to the transcript level of the internal standard gene, *Gapdh*. The primer sequences are listed in Table S2.

### Western blot

The different tissues (cerebellum, liver, bladder, tail, and cochlea), which were homogenized by a homogenizer, of wild-type and *Gjb2*^*35delG/35delG*^ mice at P14, were lysed in 1 × RIPA buffer [50 mM Tris–Cl, pH 7.4, 150 mM NaCl, 5 mM EDTA, 1% (v/v) Triton X-100, 0.5% sodium pyrophosphate, 0.1% sodium dodecyl sulfate] (Cell Signaling Technology) containing protease inhibitor cocktail (Sigma) on ice for 2 h. Before gel electrophoresis, protein lysate was boiled with 5 × loading buffer at 60 °C for 10 min. Proteins were separated by 15% SDS–PAGE and then blotted on a PVDF (polyvinylidene difluoride) membrane, blocked with blocking solution (10% non-fat dry milk in TBST buffer) for a few hours and incubated with the appropriate primary antibody (rabbit anti-CX26, 1:1000 dilution, Invitrogen, Cat#512800; mouse anti-CX30, 1:1000 dilution, SANTA CRUZ, Cat#sc-514847; and mouse anti-GAPDH, 1:5000, Proteintech, Cat#60004-1-lg) in TBST buffer overnight at 4 °C. The membranes were washed three times for 10 min each with TBST and incubated with the appropriate secondary antibody (Goat anti-rabbit IgG, 1:5000 dilution, BBI, Cat#D110058-0001 and goat anti-mouse IgG, 1:10,000 dilution, Proteintech, Cat#SA00001-1) in TBST for 1 h at room temperature. Chemiluminescence detection was performed using an ECL reagent solution kit from Share-Bio.

### Auditory testing

ABR and DPOAE measurements were recorded using an RZ6 acoustic system (Tucker-Davis Technologies, Alachua, FL, USA). Mice were anesthetized with xylazine (10 mg/kg) and ketamine (100 mg/kg) via intraperitoneal injection. For ABR measurements, three needle electrodes were inserted separately into the subcutaneous tissue of the cranial vault between the two ears (the reference electrode), the mastoid portion behind the left pinna (the recording electrode), and the dorsal rump of the animal (the grounding electrode). Each animal was stimulated with 5-ms tone pips to induce ABR potentials, followed by amplification (10,000 responses), filtering (300 Hz–3 kHz passband), and averaging (512 responses) at each sound pressure level (SPL). Tone burst acoustic stimuli were given at frequencies of 4, 8, 16, 24, and 32 kHz. The ABR threshold was determined as the lowest SPL at which an ABR wave peak could be visually detected and repeated compared to background noise. For DPOAE measurements, the f_1_ and f_2_ primary tones (*f*_2_/*f*_1_ = 1.2) were presented with f_2_ at 4, 8, 16, 24, and 32 kHz and *L*_1_ − *L*_2_ = 0 dB sound pressure level (dB SPL). For each f_2_/f_1_ primary pair, L_2_ was swept in 5 dB increments from 80 to 20 dB SPL. Waveform and spectral averaging were used at each level to increase the signal-to-noise ratio of the recorded ear-canal sound pressure. DPOAE threshold was defined from the average spectra as the 2*f*_1_–*f*_2_ level producing a DPOAE of magnitude 5 dB SPL above the noise floor. The mean noise floor level was under 0 dB across all frequencies. In general, ABR and DPOAE thresholds and amplitudes were defined by two independent observers.

### Immunofluorescence

Cochleae from E17.5, P0, 2-week-old, and 5-week-old mice were harvested and fixed with 4% paraformaldehyde at room temperature for 2 h. The cochleae of 2-week-old and 5-week-old mice were decalcified in 10% ethylenediamine tetraacetic acid (EDTA) at room temperature for 4–6 h. The entire basilar membranes from neonatal mice and adult mice were divided into the apical, middle, and basal turns according to the sensitivity to different sound frequencies. The tissue was permeabilized and then infiltrated with 1% Triton X-100 and blocked with 10% bovine serum for 12–16 h at 4 °C before primary antibody incubation. To observe the degeneration of hair cells and supporting cells, rabbit anti-Myosin VII (1:800 dilution, Proteus BioSciences, Cat#25-6790) and goat anti-human/mouse/rat Sox2 (1:500 dilution, R&D Systems, Cat#AF2018), respectively, were incubated at 4 °C overnight. Donkey-anti-rabbit Cy3 (1:500 dilution, Jackson ImmunoResearch, Cat#711-165-152) and donkey anti-goat Alexa Fluor 647 (1:500 dilution, Jackson ImmunoResearch, Cat#705-605-147) secondary antibodies were incubated in the dark at room temperature for 2 h after rinsing three times with 1 × PBS. To observe the expression of gap junction channel proteins, rabbit anti-connexin 26 (1:200 dilution, Invitrogen, Cat#512800) and mouse IgG2a anti-connexin 30 (1:400 dilution, SANTA CRUZ, Cat#sc-514847) were incubated at 4 °C overnight. Donkey-anti-rabbit Cy3 (1:500 dilution, Jackson ImmunoResearch, Cat#711-165-152) and donkey anti-mouse Alexa Fluor 488 (1:500 dilution, Jackson ImmunoResearch, Cat#AB_2340846) secondary antibodies were incubated in the dark at room temperature for 2 h after rinsing three times with 1 × PBS. To observe apoptosis, rabbit cleaved caspase-3 antibody (1:500 dilution, Cell Signaling Technology, Cat#9664S) was incubated at 4 °C overnight, and donkey-anti-rabbit Cy3 secondary antibody (1:500 dilution, Jackson ImmunoResearch, Cat#711-165-152) was incubated in the dark at room temperature for 2 h after rinsing three times with 1 × PBS. DAPI was used as a nuclear counterstain (1:1000 dilution, Sigma, Cat#D9542). Fluorescent Z-stack images were collected using a Leica TCS SP8 laser scanning confocal microscope and a 40 × 1.30 or 63 × 1.40 glycerol immersion objective. The maximum intensity projections are shown in the figures.

### Cell counting

For cell counting, the numbers of Myo7a^+^ hair cells, Sox2^+^ supporting cells, including three rows of Deiters’ cells and one row of outer/inner pillar cells [62], were manually counted in every 100-μm region of the apical, middle, and basal turns of the cochlea using Leica TCS SP8 software. The results are presented as the mean ± s.e.m.

### Scanning electron microscopy (SEM)

Cochleae from E17.5 and P0 mice were harvested and fixed in 2.5% glutaraldehyde at 4 °C overnight, then decalcified in 10% ethylenediamine tetraacetic acid (EDTA) at room temperature for 4–6 h and dissected. The dissected tissues were fixed with 1% osmium tetraoxide (OsO_4_) at 4 °C for 2 h and dehydrated in an increasing ethanol series and pentyl acetate, and then dried in an HCP-2 critical point desiccator for 2 h. SEM images were obtained with a high vacuum field emission scanning electron microscope (Hitachi SU-8010) at 3.5 kV (low magnification) or 10.0 kV (high magnification).

### Resin sections and transmission electron microscopy (TEM)

Cochleae from 2-week-old mice were harvested and fixed in 2.5% glutaraldehyde at 4 °C overnight, and decalcified in 10% ethylenediamine tetraacetic acid (EDTA) at room temperature for 4–6 h. After decalcification, the cochleae were placed with 2.5% glutaraldehyde and fixed with 1% osmium tetraoxide (OsO_4_) at 4 °C for 2 h. Then, the tissues were dehydrated in an increasing ethanol series and acetone prior to incubation in a dilution of liquid epoxy resin and acetone at a ratio of 1/2 at room temperature for 3 h. Then, a mixture of 67% epoxy resin and 33% acetone replaced the liquid at room temperature overnight. Subsequently, tissue samples were incubated in pure epoxy resin at 37 °C for 2–3 h. The specimens were subsequently transferred to an embedding mold and labeled followed by incubation in a drying oven at 37 °C, 45 °C, and 60 °C for overnight, 12 h, and 24 h, respectively. The samples were sectioned (0.8 μm in thickness) on a LEICA EM TRIM2 and stained with Toluidine Blue (89,640-5G; Sigma-Aldrich, USA) at 60 °C for light microscopy observation with a magnification of 100 × and 400 ×. For TEM analysis, specimens were then trimmed and ultrathin sections (70 nm in thickness) were acquired on a Leica EM UC7 and stained with 3% uranium acetate-lead citrate. TEM images were obtained with transmission electron microscopy (PHILIPS CM-120) at 460 × and 1400 × magnification.

### RNA-seq and data analysis

The cochleae of wild-type and *Gjb2*^35delG/35delG^ mice were carefully collected and surgically divided into three equal sections, including the apical, middle, and basal turns. The total RNA of these sections was collected using a miRNeasy Micro Kit (QIAGEN, Cat#217084) following the manufacturer’s instructions. All collected RNA was used for library construction with the VAHTS Universal V8 RNA-seq Library Prep Kit for Illumina (Vazyme, Cat# NR605). RNA-seq libraries were subjected to deep sequencing on an Illumina NovaSeq6000 platform following the manufacturer’s instructions (Illumina) to produce 150 bp paired-end reads by Genergy Biotechnology Co. Ltd. (Shanghai, China). Raw RNA-seq reads were cleaned using fastp (v0.20.0) and aligned to mm10 using STAR (v2.7.3a). Only unique mapped reads were kept and counted by featureCounts (v2.0.0), and FPKM (Fragments Per Kilobase of transcript per Million mapped fragments) and TPM (Transcripts Per Kilobase Million) were calculated by stringtie (v2.0). Genome coverage was counted by bamCoverage (v3.3.1). Differential gene expression was determined using DESeq2 (v1.30.0), and clusterProfiler (v3.18.1) was used for GO and KEGG analysis in R.

### Endocochlear potential (EP)

EP was recorded in mice at P14 and P35. Mice were anesthetized with xylazine (10 mg/kg) and ketamine (100 mg/kg) via intraperitoneal injection and placed on a heating pad to maintain body temperature. Hair on the neck and anterior chest of anesthetized mice was removed. The trachea was dissected to maintain a normal airway, and soft tissues including muscle, fat, and vessels were ligated and separated away from the bulla. Some of the bulla wall was removed to allow clear visualization of the cochlea, and a small opening was made over the stria vascularis in the middle turn of the cochlea. A glass micropipette filled with 150 mM KCl was connected to a capacity-compensated direct-current preamplifier, and a ground electrode was inserted into the chest muscle. The micropipette was attached to a micro-manipulator and slowly advanced through the lateral wall into the scala media. Amplified electrical signals were recorded through an EPC10/2 amplifier (HEKA, Lambrecht/Pfalz, Germany) driven by a PC running Patchmaster (HEKA). The EP was calculated as the maximum change in voltage as the micropipette tip passed from the lateral wall tissue into the endolymph of the scale media [56].

### Statistics

For all figures, results are shown as the average mean ± standard error of the mean (s.e.m). Statistical testing was performed using GraphPad Prism 7, and significance was determined by two-tailed, unpaired Student’s t-test. *P < 0.05; **P < 0.01; ***P < 0.001; n.s, no significant difference.

## Supplementary Information

Below is the link to the electronic supplementary material.

Supplementary file1 (DOCX 3839 KB)

## Data Availability

Raw and processed RNA-seq data have been deposited in the Gene Expression Omnibus (GEO) under accession number GSE215106.
