# Supplementary material for: The pathogenesis of common Gjb2 mutations associated with human hereditary deafness in mice
Source: Cell Mol Life Sci. 2023 May 13;80(6):148. doi: 10.1007/s00018-023-04794-9 (PMC10182940; doi:10.1007/s00018-023-04794-9)
Supplement: Supplementary file 1 — Supplementary file1 (DOCX 3839 KB) [file 18_2023_4794_MOESM1_ESM.docx]

**Supplementary information for**

**The pathogenesis of common *Gjb2* mutations associated with human hereditary deafness in mice**

Qing Li^1,8,*^ **·** Chong Cui^2,3,4,8^ **·** Rongyu Liao^1,9^ **·** Xidi Yin^1,9^ **·** Daqi Wang^2,3,4,9^ **·** Yanbo Cheng^5^ **·** Bowei Huang^2,3,4^ **·** Liqin Wang^2,4^ **·** Meng Yan^6^ **·** Jinan Zhou^2,3,4^ **·** Jingjing Zhao^2,4^ **·** Wei Tang^1^ **·** Yingyi Wang^5^ **·** Xiaohan Wang^7^ **·** Jun Lv^2,3,4^ **·** Jinsong Li^1,5,6^ **·** Huawei Li^2,3,4,*^ and Yilai Shu^2,3,4,*^

^1^State Key Laboratory of Cell Biology, Shanghai Key Laboratory of Molecular Andrology, Shanghai Institute of Biochemistry and Cell Biology, Center for Excellence in Molecular Cell Science, Chinese Academy of Sciences, Shanghai, China.

^2^ENT institute and Department of Otorhinolaryngology, Eye & ENT Hospital, State Key Laboratory of Medical Neurobiology and MOE Frontiers Center for Brain Science, Fudan University, Shanghai, China.

^3^Institutes of Biomedical Sciences, Fudan University, Shanghai, China.

^4^NHC Key Laboratory of Hearing Medicine, Fudan University, Shanghai, China.

^5^School of Life Science and Technology, Shanghai Tech University, Shanghai, China.

^6^School of Life Science, Hangzhou Institute for Advanced Study, University of Chinese Academy of Sciences, Hangzhou, China.

^7^Renerval Biotherapeutics, Jiangsu, China.

^8^Q. Li, and C. Cui, contributed equally to this work.

^9^These authors contributed equally to this work.

^*^Corresponding authors: Yilai Shu: [yilai_shu@fudan.edu.cn](mailto:yilai_shu@fudan.edu.cn).; Huawei Li: [hwli@shmu.edu.cn](mailto:hwli@shmu.edu.cn); Qing Li: [liqing2015@sibcb.ac.cn](mailto:liqing2015@sibcb.ac.cn).

**This PDF file includes:**

Figures. S1 to S9

Tables. S1 to S2

**Supplementary Figures**


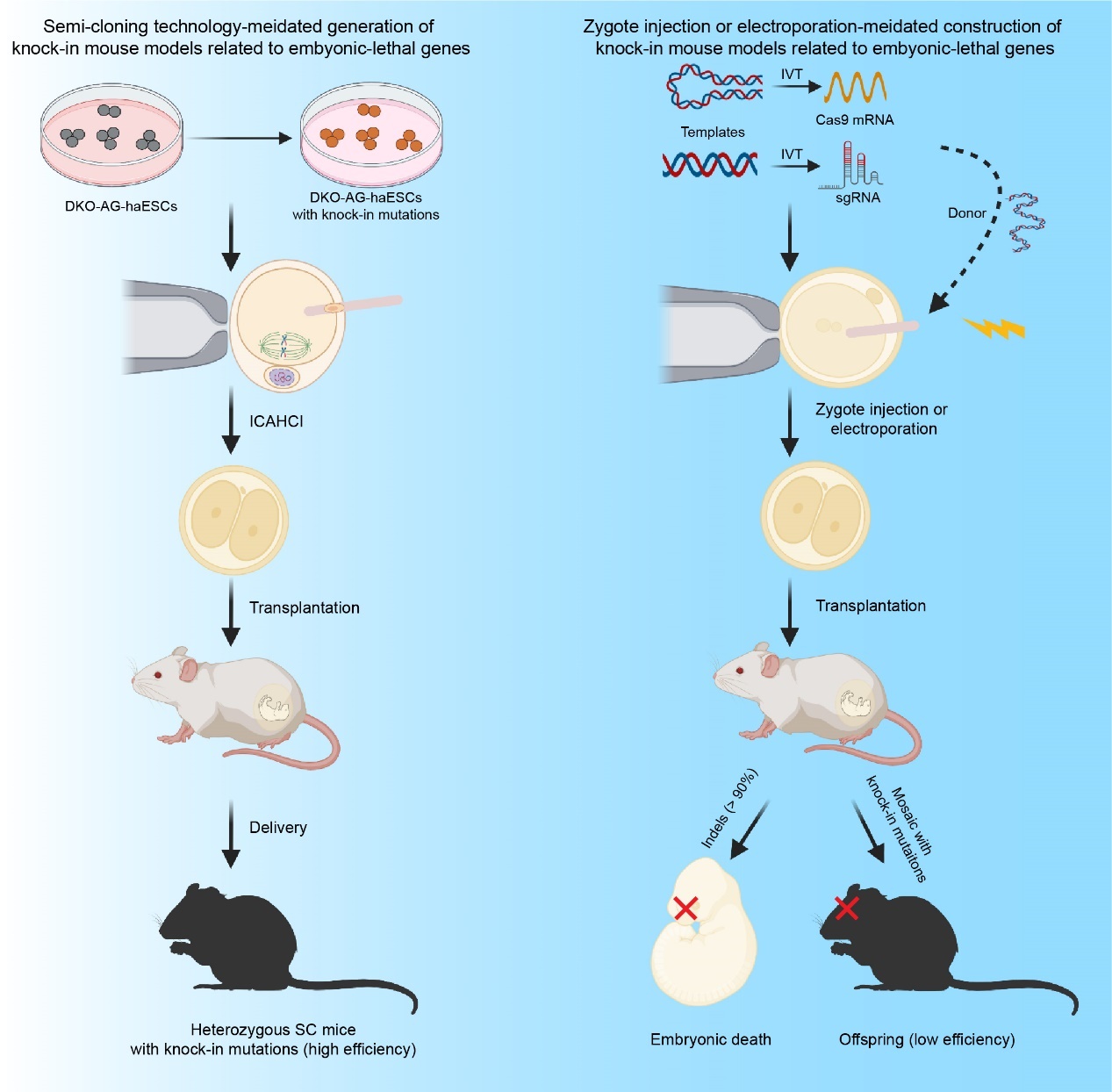


**Figure S1. Comparison of semi-cloning technology and zygote injection/electroporation-mediated construction of knock-in mouse models of embryonic-lethal genes (created in**[**BioRender.com**](http://biorender.com/)**).**


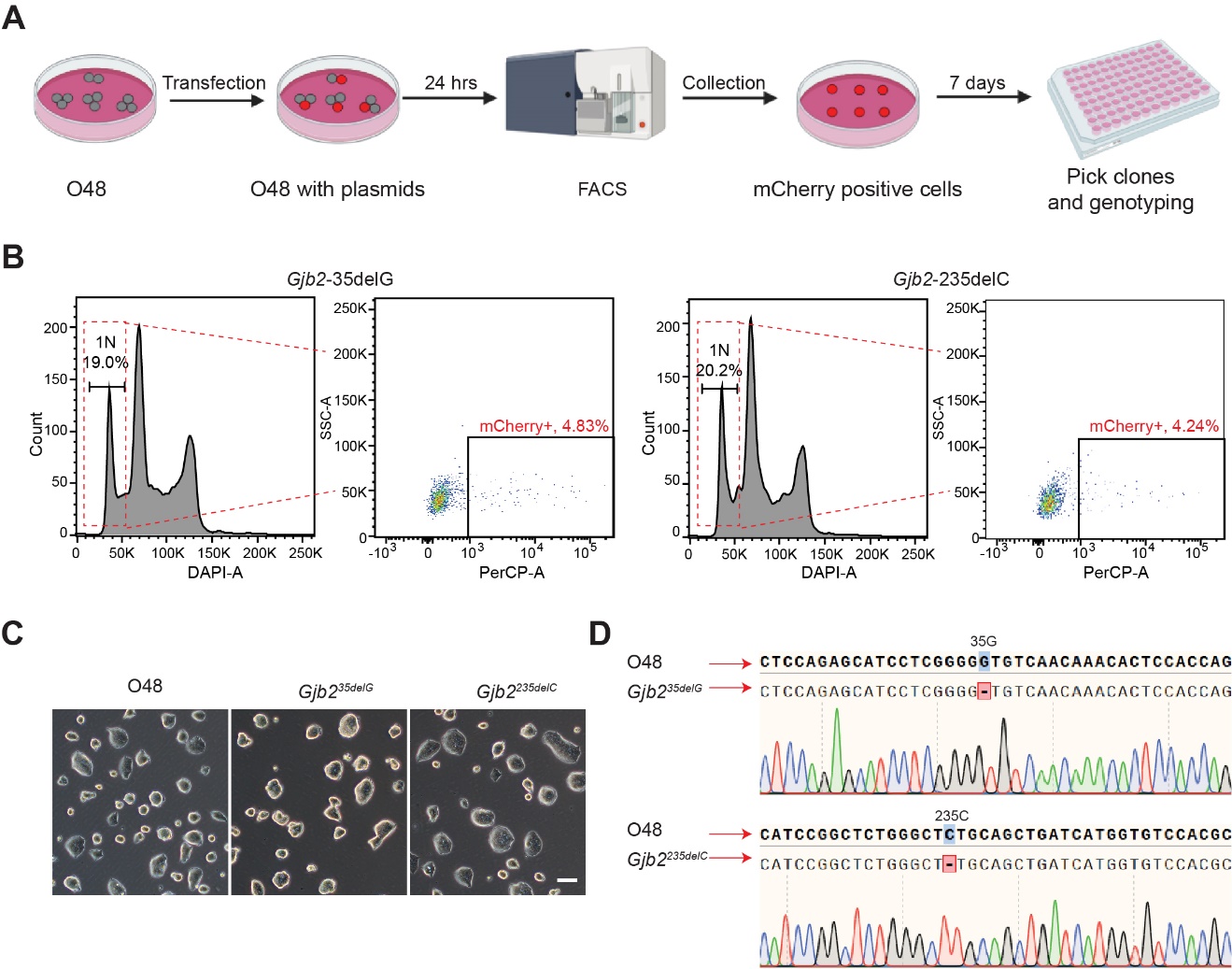


**Figure S2. Generation of DKO-AG-haESCs carrying 35delG and 235delC in *Gjb2*.** (*A*) Experimental procedures for generating DKO-AG-haESCs carrying 35delG and 235delC in *Gjb2* *via* CRISPR-Cas9 technology. (*B*) O48 cells with haploid genome and highly expressed mCherry were enriched through FACS after transfection, followed by single-cell expansion to obtain *Gjb2^35deG^* and *Gjb2^235delC^* cell clones. (*C*) Images of cultured O48, *Gjb2^35deG^*, and *Gjb2^235delC^* haploid ESCs. Scale bar, 100 μm. (*D*) Representative Sanger sequencing results of *Gjb2^35deG^* and *Gjb2^235delC^* haploid ESCs.


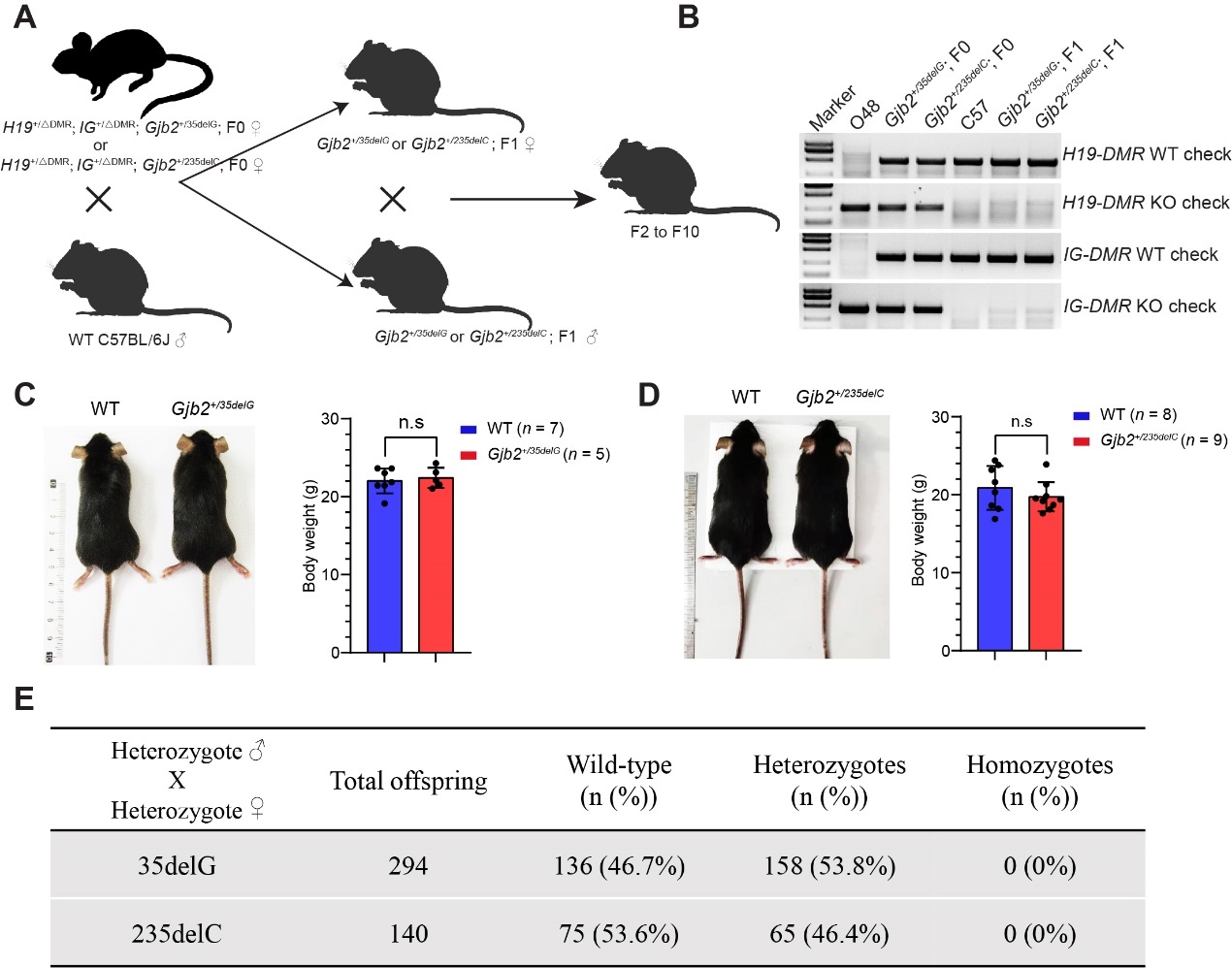


**Figure S3.** **35delG and 235delC heterozygotes showed normal growth but homozygous lethality.** (*A*) Schematic diagram of backcrossing of *Gjb2^+/35deG^* and *Gjb2^+/235delC^* F0 mice with C57BL/6J mice to generate the F2 to F10 mice. (*B*) Genotyping of *H19*-DMR and *IG*-DMR in the offspring of *Gjb2^+/35deG^* and *Gjb2^+/235delC^* mice. Mice with *H19*-DMR or *IG*-DMR deletion were abandoned in the F2 generation. (*C*) Representative images (left) and body weight (right) of wild-type and *Gjb2^+/35delG^* males at 8 weeks of age. (*D*) Representative images (left) and body weight (right) of wild-type and *Gjb2^+/235delC^* males at 8 weeks of age. Data are the mean ± s.e.m of the indicated biological replicates in (*C*) and (*D*). n.s, no significance. (*E*) Summary of the genotypes of offspring of 35delG and 235delC heterozygotes. The absence of homozygotes indicated the homozygous lethality of *Gjb2^35delG/35delG^* and *Gjb2^235delC/235delC^*.


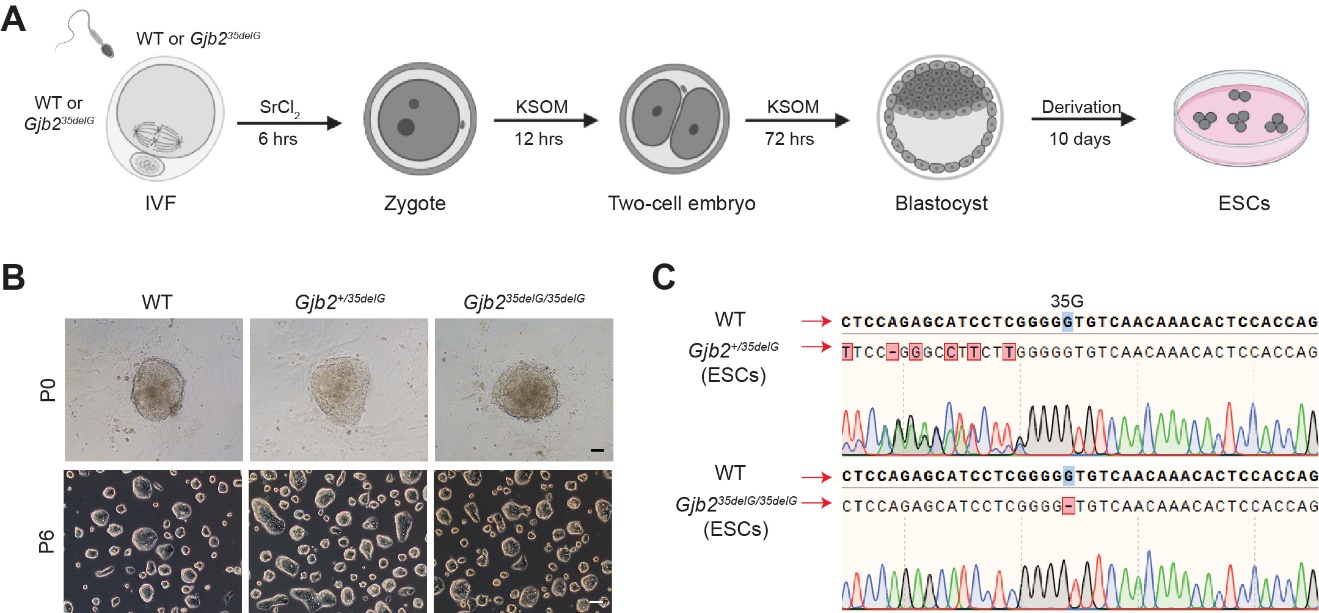


**Figure S4. The derivation of the ESC line carrying homozygous 35delG in *Gjb2*.** (*A*) Schematic diagram of the generation of *Gjb2* 35delG homozygous ESCs from blastocysts (created in [BioRender.com](http://biorender.com/)). (*B*) Cell morphology of cultured wild-type, *Gjb2^+/35delG^*, and *Gjb2^35delG /35delG^* ESCs at passage 0 (P0) and P6. Scale bar, 100 μm. (*C*) Representative Sanger sequencing results of *Gjb2^+/35deG^* and *Gjb2^35del /35delG^* ESCs.


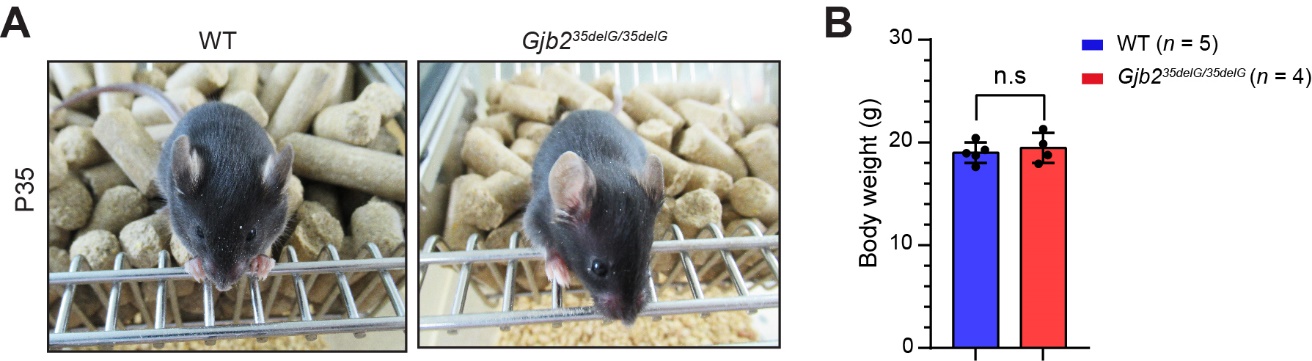


**Figure S5.** **35delG homozygous mice showed normal growth at P35**. (*A*) Representative images of wild-type and *Gjb2^35delG/35delG^* males at P35. (*B*) The body weight of wild-type and *Gjb2^35delG/35delG^* males at P35, indicating that the inactivation of *Gjb2* did not influence individual growth. Data are the mean ± s.e.m of the indicated biological replicates. n.s, no significance.


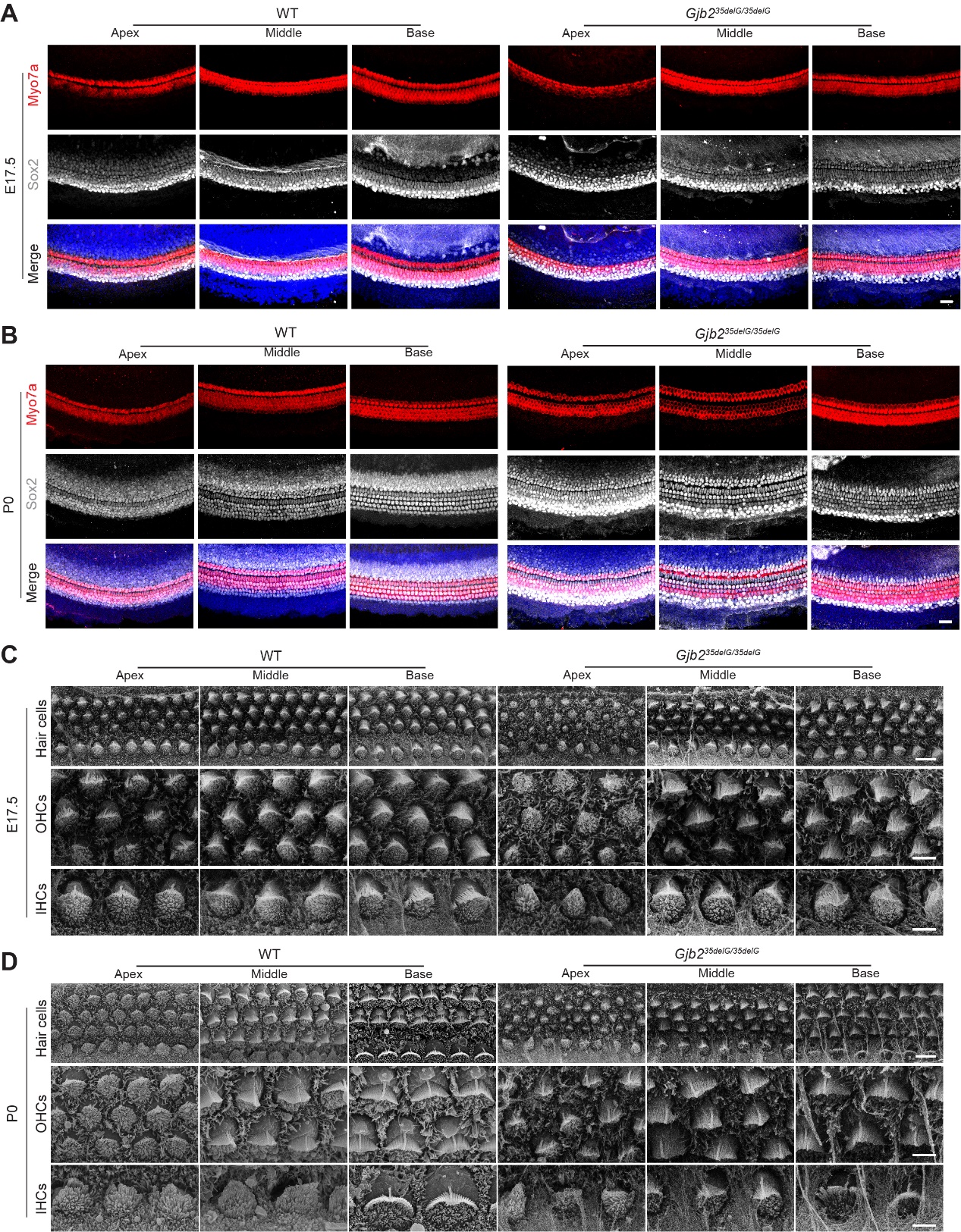


**Figure S6.** ***Gjb2* is dispensable for the development of hair cells and supporting cells in the cochlea at prenatal stages.** (*A and B*) Representative confocal microscopy images of Myo7a and Sox2 immunofluorescence in the apical, middle, and basal turns of the cochleae from wild-type and *Gjb2^35delG/35delG^* mice at E17.5 (*A*) and P0 (*B*). Three independent mice were analyzed in each group. Scale bar, 20 μm. (*C and D*) Representative SEM images of hair cell stereocilia morphology in the apical, middle, and basal turns of the cochleae from wild-type and *Gjb2^35delG/35delG^* mice at E17.5 (*C*) and P0 (*D*)*.* Low magnification: hair cells in the upper panel*.* Scale bar, 50 μm. High magnification: IHCs in the middle panel and OHCs in the bottom panel. Scale bar, 25 μm. Three independent mice were analyzed in each group.


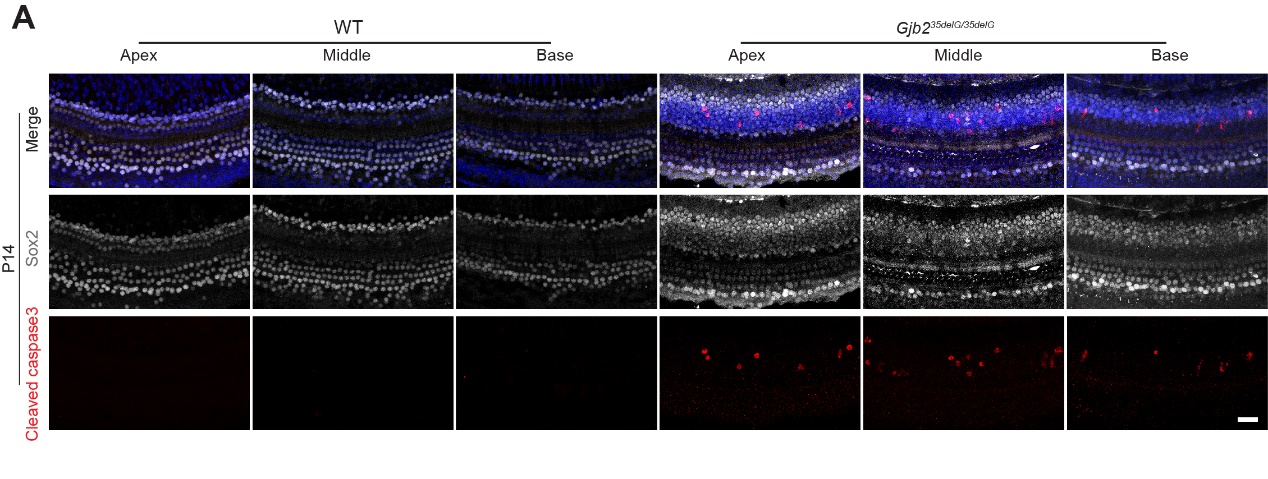


**Figure S7.** **The influence of GJB2 absence on supporting cells.** (*A*) The representative confocal microscopy images of Caspase-3 immunofluorescence in the apical, middle, and basal turns of the cochleae from wild-type and *Gjb2^35delG/35delG^* mice at P14, indicating a small number of apoptotic cells were seen in the greater epithelial ridge (GER). Three independent mice were analyzed for each group. Scale bar, 20 μm.


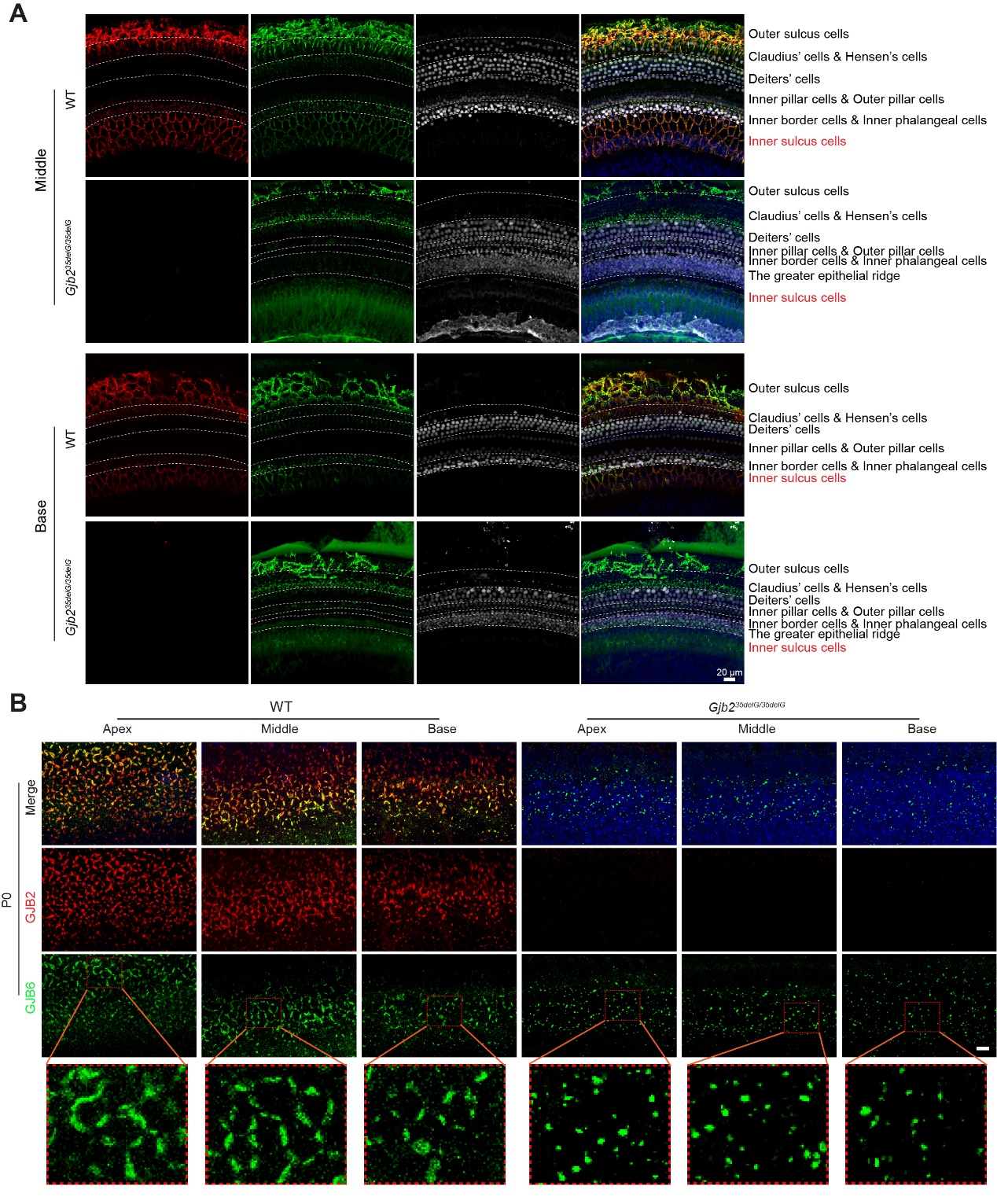


**Figure S8.** **The influence of GJB2 absence on GJCs.** (*A*) The panoramic view of representative confocal images of middle and basal turns from wild-type and 35delG homozygous mice at P14, by staining GJB2, GJB6, and Sox2. The regions containing different supporting cells were delineated in the figure. Scale bar, 20 μm. (*B*) Representative confocal microscopy images of GJB2 and GJB6 immunofluorescence in the apical, middle, and basal turns of the cochleae from wild-type and *Gjb2^35delG/35delG^* mice at P0. Three independent mice were analyzed for each group. Scale bar, 20 μm.


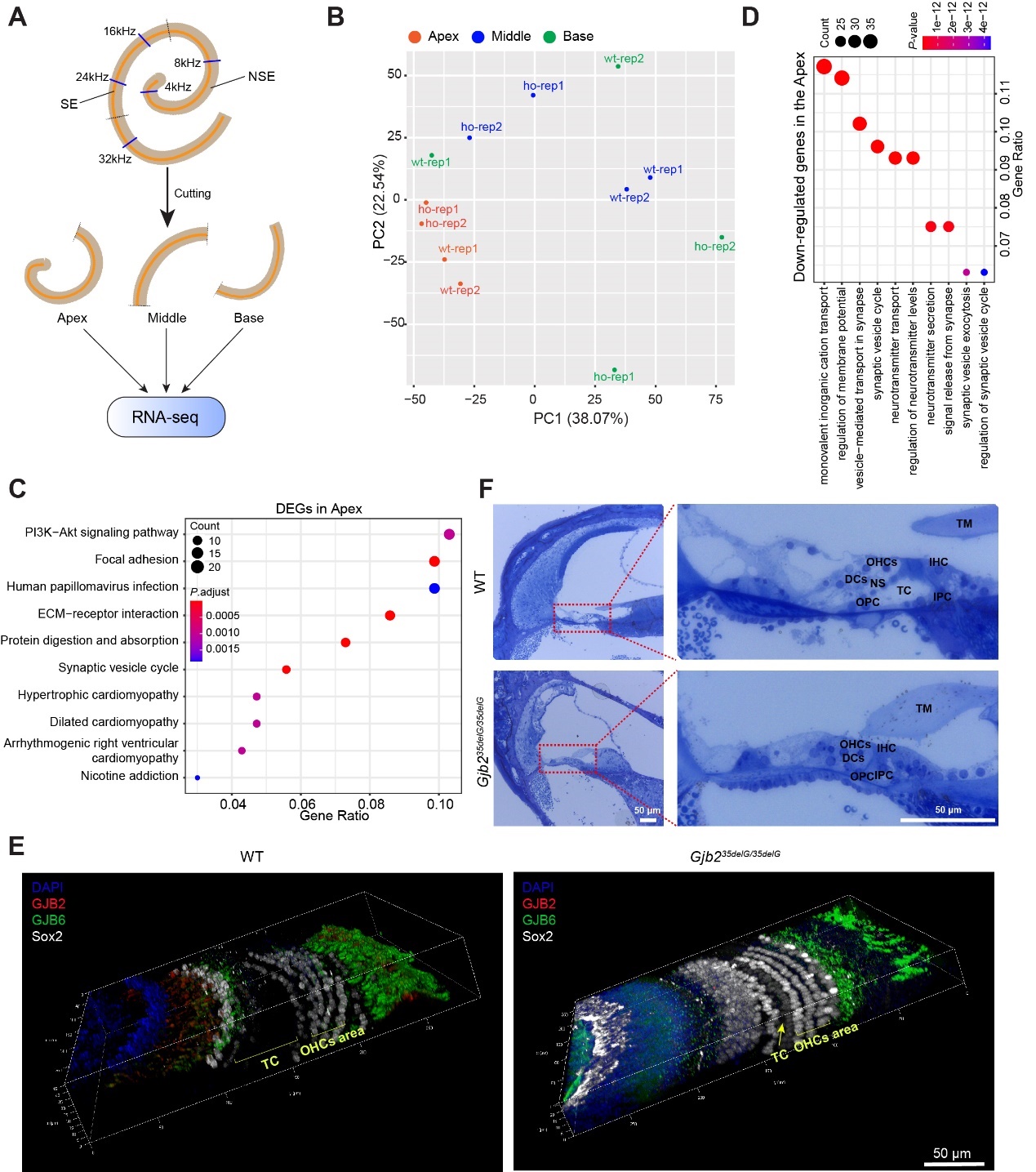


**Figure S9.** **The impairment of cochleae in 35delG homozygous mice.** (*A*) Schematic diagram of the isolation and RNA-seq of cochleae from wild-type and *Gjb2^35deG /35deG^* mice at P14. Cochleae were divided into three equal sections referred to as the apical, middle, and basal turns according to the sensitivity of different sound frequencies. SE, sensory epithelium; NSE, non-sensory epithelium. (*B*) The principal component analysis of RNA-seq data from wild-type and *Gjb2^35delG/35delG^* cochleae, including the apical, middle, and basal turns. Two biological repeats for each group. (*C*) The KEGG pathway analysis of DEGs between wild-type and *Gjb2*^3^*^5delG/35delG^* cochleae in the apical turn. (*D*) GO analysis of down-regulated genes between wild-type and *Gjb2^35delG/35delG^* in the apical turn. (*E*) The three-dimensional analysis of the tunnel of Corti at the middle turn of the basilar membrane in wild-type and *Gjb2^35delG/35delG^* cochleae. The three-dimensional images were constructed with the confocal images in Figure. S8A. TC, tunnel of Corti; OHCs, outer hair cells. Scale bar, 50 μm. (*F*) A full view of the tunnel of Corti from wild-type and *Gjb2^35delG/35delG^* mice at P14. The right images show the high magnification (400x magnification) views of the left red box (100x magnification). Two independent mice were analyzed for each group. Scale bar: 50 μm. TC, tunnel of Corti; NS, Nuel's space; IHC, inner hair cell; OHCs, outer hair cells; IPC, inner pillar cell; OPC, outer pillar cell; DCs, Deiters’ cells; TM: tectorial membrane.

| **ES cell lines** | **No. of injected embryos transferred (mixed wild-type 2N embryos)** | **No. of recipient mice** | **No. total born (%)** | **No. pups alive at term (% transferred embryos)** | **No. pups surviving more than 2-weeks (35delG homozygotes)** |
| --- | --- | --- | --- | --- | --- |
| *Gjb2^35delG/35delG^*-2 P6 | 90 | 3 | 7 (7.8) | 4 (4.4) | 0 (0) |
| *Gjb2^35delG/35delG^*-3 P5 | 96 | 4 | 9 (9.4) | 8 (8.3) | 2 (2) |
| *Gjb2^35delG/35delG^*-3 P6 | 60 | 2 | 9 (15) | 8 (13.3) | 0 (0) |
| *Gjb2^35delG/35delG^*-3 P6 | 88 | 4 | 11 (12.5) | 9 (10.2) | 1 (1) |
| *Gjb2^35delG/35delG^*-3 P6 | 22 | 1 | 4 (18.2) | 4 (18.2) | 2 (2) |
| *Gjb2^35delG/35delG^*-3 P6 | 92 | 4 | 9 (9.8) | 5 (5.4) | 0 (0) |
| *Gjb2^35delG/35delG^*-3 P7 | 88 | 4 | 12 (13.6) | 9 (10.2) | 0 (0) |
| *Gjb2^35delG/35delG^*-3 P7 | 88 | 4 | 6 (6.9) | 2 (2.3) | 0 (0) |
| *Gjb2^35delG/35delG^*-3 P8 | 80 | 4 | 8 (10) | 6 (7.5) | 0 (0) |
| *Gjb2^35delG/35delG^*-3 P8 | 105 | 5 | 8 (7.6) | 4 (3.8) | 1 (1) |
| *Gjb2^35delG/35delG^*-3 P6 | 66 | 3 | 5 (4.5) | 1 (1.5) | 1 (1) |
| *Gjb2^35delG/35delG^*-3 P7 | 132 | 6 | 19 (14.4) | 15 (11.4) | 8 (8) |
| *Gjb2^35delG/35delG^*-3 P8 | 120 | 5 | 10 (8.3) | 7 (5.8) | 1 (1) |
| *Gjb2^35delG/35delG^*-3 P8 | 120 | 5 | 16 (13.3) | 12 (10) | 5 (5) |
| *Gjb2^35delG/35delG^*-3 P9 | 156 | 6 | 26 (16.7) | 22 (14.1) | 4 (4) |
| *Gjb2^35delG/35delG^*-6 P5 | 156 (48)^a^ | 6 | 27 (17.3) | 26 (16.7) | 20 (1) |
| *Gjb2^35delG/35delG^*-6 P6 | 132 (36)^a^ | 6 | 24 (18.2) | 24 (18.2) | 22 (0) |
| *Gjb2^35delG/35delG^*-6 P6 | 154 (42)^a^ | 7 | 23 (14.9) | 23 (14.9) | 18 (0) |
| ^a^ Transferred tetraploid embryos mixed with some wild-type diploid embryos in each uterus of recipients. | | | | |  |

**Table S1.** Summary of homozygous 35delG mice generation *via* tetraploid embryo complementation.

**Table S2.** List of oligo and primer sequence information related to experimental procedures.

| **Name** | **Sequence (5’-3’)** | **Application** |
| --- | --- | --- |
| Gjb2-35delG-oligoF | CACCAGTGTTTGTTGACACCCCCG | Oligos for construction of pX330-mCherry-35delG and pX330-mCherry-235delC |
| Gjb2-35delG-oligoR | AAACCGGGGGTGTCAACAAACACT |  |
| Gjb2-235delC-oligoF | CACCCTGGGCTCTGCAGCTGATCA |  |
| Gjb2-235delC-oligoR | AAACTGATCAGCTGCAGAGCCCAG |  |
| Gjb2-35/235-leftF | GCCCTGTTCCTCTCACACAG | Primers for construction of 19T-Gjb2-35G and 19T-Gjb2-235C |
| Gjb2-35-leftR | CTGGTGGAGTGTTTGTTGACACCCCGAGG |  |
| Gjb2-35-rightF | CCAGAGCATCCTCGGGGTGTCAACAAACACT |  |
| Gjb2-35/235-rightR | CTCAGTGGCACCTTCCTCAG |  |
| Gjb2-235-leftR | TGGCGTGGACACCATGATCAGCTGCAAGCCCA |  |
| Gjb2-235-rightF | TCCGGCTCTGGGCTTGCAGCTGATCATGGTGTC |  |
| Gjb2-checkF | TGCCCGGGAAGACAGTTAAG | Genotyping |
| Gjb2-checkR | TTCATGTCTCCGGTAGGCCA |  |
| H19-DMR-wt-F | AGATGGGGTCATTCTTTTCC |  |
| H19-DMR-wt-R | ATTGCTCTTAGCTTCTGTTG |  |
| H19-DMR-del-F | GTGGTTAGTTCTATATGGGG |  |
| H19-DMR-del-R | TCTTACAGTCTGGTCTTGGT |  |
| IG-DMR-wt-F | TGTATGGTACAAACCGGGTGA |  |
| IG-DMR-wt-R | ACTTTTGCTGTGAGGCACTTG |  |
| IG-DMR-del-F | TGTGCAGCAGCAAAGCTAAG |  |
| IG-DMR-del-R | ATACGATACGGCAACCAACG |  |
| Gapdh-qPCR-F | CACTCTTCCACCTTCGATGC | Real time PCR |
| Gapdh-qPCR-R | CTCTTGCTCAGTGTCCTTGC |  |
| Gjb2-qPCR-F | ATTTCGGACCAACCCAGG |  |
| Gjb2-qPCR -R | CCCAATCCATCTTGTCCTCTG |  |
| Gjb6-qPCR -F | GAAGCCCTGGAGAACAAGAC |  |
| Gjb6-qPCR -R | CATGACTCGGAAAATAAAGATGACC |  |
